# Molecular Profiling Defines Evolutionarily Conserved Transcription Factor Signatures of Major Vestibulospinal Neuron Groups

**DOI:** 10.1523/ENEURO.0475-18.2019

**Published:** 2019-02-27

**Authors:** Anders Lunde, Benjamin W. Okaty, Susan M. Dymecki, Joel C. Glover

**Affiliations:** 1 Department of Molecular Medicine, University of Oslo, Oslo 0372, Norway; 2 Department of Genetics, Harvard Medical School, Boston, Massachusettes 02115; 3 Norwegian Center for Stem Cell Research, Department of Immunology and Transfusion Medicine, Oslo University Hospital, Oslo 0372, Norway

**Keywords:** brainstem, hindbrain, projection, projection neurons, vestibulospinal

## Abstract

Vestibulospinal neurons are organized into discrete groups projecting from brainstem to spinal cord, enabling vertebrates to maintain proper balance and posture. The two largest groups are the lateral vestibulospinal tract (LVST) group and the contralateral medial vestibulospinal tract (cMVST) group, with different projection lateralities and functional roles. In search of a molecular basis for these differences, we performed RNA sequencing on LVST and cMVST neurons from mouse and chicken embryos followed by immunohistofluorescence validation. Focusing on transcription factor (TF)-encoding genes, we identified TF signatures that uniquely distinguish the LVST from the cMVST group and further parse different rhombomere-derived portions comprising the cMVST group. Immunohistofluorescence assessment of the CNS from spinal cord to cortex demonstrated that these TF signatures are restricted to the respective vestibulospinal groups and some neurons in their immediate vicinity. Collectively, these results link the combinatorial expression of TFs to developmental and functional subdivisions within the vestibulospinal system.

## Significance Statement

The molecular underpinnings of hodological and functional subdivisions within brainstem-to-spinal cord projection neurons are poorly understood. Transcriptomic profiling is an important step toward obtaining a molecular characterization of individual projection neuron groups and identifying candidate genes potentially involved in their specification. Here we use this approach to identify transcription factor signatures conserved in mouse and chicken that distinguish the two major vestibulospinal projection neuron groups and define coherent subpopulations within them, using whole-transcriptome sequencing and immunohistofluorescence combined with retrograde tracing.

## Introduction

Projection neurons in the brainstem represent the major source of descending inputs to spinal circuits and comprise a large set of neuron groups with diverse functions. Their original anatomic classification led to the definition of projection systems such as the reticulospinal, vestibulospinal, tectospinal, and rubrospinal. Many of these are markedly heterogeneous, composed of distinct subpopulations with differing projection pathways, spinal targets, and functional roles. How this diverse collection of projection neuron groups arises and collectively regulates spinal function is poorly understood. A key element in understanding this lies in the molecular programs that specify neuron groups within each projection system. A first step toward elucidating such programs is to identify molecular signatures that distinguish the different neuron subpopulations.

The vestibulospinal (VS) projection is one of the better characterized brainstem projection systems, and plays a central role in the control of posture and movement. VS neurons receive direct and indirect input from the peripheral vestibular organs, and innervate alpha motoneurons in the spinal cord monosynaptically and polysynaptically, permitting rapid regulation of musculature in response to movements of the head and body (Grillner et al., 1970; Akaike, 1983; Uchino and Kushiro, 2011; Di Bonito et al., 2015; Kasumacic et al., 2015; Murray et al., 2018). VS neurons also receive inputs from the cerebellum and from other brainstem regions through which their activity can be integrated more broadly into ongoing motor control.

The classical description of the VS system has included the unilaterally projecting lateral vestibulospinal tract (LVST) and the bilaterally-projecting medial vestibulospinal tract (MVST). We showed earlier that the LVST derives from the ipsilaterally-projecting LVST neuron group and the MVST derives from the distinct ipsilaterally-projecting iMVST and contralaterally-projecting cMVST neuron groups (Glover and Petursdottir, 1988, 1991; Díaz et al., 1998, 2003; Pasqualetti et al., 2007; Kasumacic et al., 2010; Di Bonito et al., 2015). These groups differ not only in axonal trajectory but also in developmental origins, locations and spinal termination patterns. The LVST neuron group derives from hindbrain rhombomere (r)4, lies primarily within the lateral vestibular nucleus, and projects along an initially lateral trajectory within the hindbrain and then down the length of the spinal cord. The iMVST neuron group derives from r6 and lies primarily within the descending vestibular nucleus, whereas the cMVST neuron group derives from r4 and r5 and overlaps the lateral, medial and descending vestibular nuclei. Both project in the medial longitudinal fascicle (MLF; respectively, ipsilaterally and contralaterally) to cervical and upper thoracic spinal segments. Axons from all three groups synapse on motoneurons and premotor interneurons, with clear examples of differential targeting (Shinoda et al., 2006; Kasumacic et al., 2010, 2015). A fourth VS group, originating from the more caudal portion of the posterior vestibular nucleus, is less well characterized (Peterson and Coulter, 1977; Peterson et al., 1978; Donevan et al., 1992).

The LVST and cMVST neuron groups are the largest of the VS groups and the most clearly conserved across the vertebrate radiation, having been described in mammals, birds, amphibians, and fish (mouse: Pasqualetti et al., 2007; chicken: Glover and Petursdottir, 1988; frog: Straka et al., 2001; fish: Glover and Fraser, unpublished observations; Suwa et al., 1996). Their different anteroposterior and dorsoventral origins, axon trajectories, and termination patterns suggest that they are genetically programmed by specific profiles of transcription factor (TF) expression (Auclair et al., 1999; Glover, 2000b; Cepeda-Nieto et al., 2005). An initial effort at profiling the expression of TFs in VS neurons was made by Chen et al. (2012), who demonstrated that the TFs Lbx1 and Phox2a/b are expressed by a population of VS neurons located in the lateral vestibular nucleus (LVN) of the mouse during embryonic development. The LVN contains the LVST group and also portions of the cMVST group and is thus hodologically heterogeneous (Díaz et al., 2003). To better define how TF expression relates to these VS groups, more comprehensive expression analysis is necessary.

Here, we use whole-transcriptome sequencing of LVST and cMVST neurons isolated from mouse and chicken embryos, followed by immunohistochemical validation, to define specific TF profiles distinguishing the LVST and cMVST groups. Immunohistochemical assessment from spinal cord to cortex shows that these TF signatures are restricted to the LVST and cMVST groups plus a few vicinal neurons. Additional TFs define spatially coherent subpopulations within these groups. These results expand our understanding of the molecular identity of VS neuron groups, provide a first step toward unraveling transcriptional heterogeneity within them, and suggest testable hypotheses about potential fate-specifying programs.

## Materials and Methods

### Animal handling and dissection

All animal procedures were approved by the Norwegian Animal Research Authority (Forsøksdyrutvalget, FDU ID 8473) and performed in accordance with the University of Oslo animal care committee's regulations and followed the Federation of European Laboratory Animal Science Associations (FELASA) guidelines. In compliance with these regulations, all efforts were made to minimize the number of mice used and their suffering. Unless otherwise noted, experiments were performed on mice of either sex from the Crl:CD1(ICR) line (RRID:IMSR_CRL:22). For r4 lineage tracing, the *b1r4-Cre* transgenic line (Di Bonito et al., 2013), which expresses c*re recombinase* exclusively in r4 under the control of the *Hoxb1* r4 enhancer (Studer et al., 1994), was used in combination with the *Ai14* Cre reporter line harboring a loxP-flanked STOP cassette preventing transcription of a CAG promoter-driven red fluorescent protein variant (tdTomato; strain 7914, Jackson Laboratories), both of either sex. The morning of vaginal plug observation was defined as embryonic day (E)0.5. Pregnant dams were anesthetized with isoflurane before cervical dislocation. Dissected embryos were kept in ice-cold (4°C), oxygenated (95% O_2_-5% CO_2_), artificial CSF [ACSF; containing the following (in mm): 128 NaCl, 3 KCl, 11 d-glucose, 2.5 CaCl_2_, 1 MgSO_4_, 1.2 NaH_2_PO_4_, 5 HEPES, and 25 NaHCO3]. Brainstems with cervical spinal cord were dissected out in cold ACSF under a dissection microscope.

Fertilized Ross II chicken eggs of either sex, acquired from Nortura, were stored at 14°C, and incubated at 37.5°C in a humidified forced draft incubator, counting start of incubation as incubation day (d)0. On the day of dissection, eggs were cracked open and embryos transferred to ice-cold (4°C), oxygenated (100% O_2_), chicken ringer solution containing the following (in mm): 137 NaCl, 5 KCl, 11 d-glucose, 2 CaCl_2_, 1 MgSO_4_, 1 NaPO_4_ buffer, pH 7.4, and 5 HEPES. Embryo stage was determined according to Hamburger and Hamilton (1992), with d7.5 and d9 corresponding to stages HH30 and HH35, respectively. Brainstems with cervical spinal cord attached were dissected out under a dissection microscope.

### Retrograde labeling with conjugated dextran amines

Isolation of LVST and cMVST neurons for RNA sequencing requires that they first be selectively retrogradely labeled via their axons to the spinal cord. To determine the earliest stages at which reliable retrograde labeling of LVST and cMVST neurons could be obtained, we performed such labeling at different stages and anteroposterior levels in mouse and chicken embryos. We found that the LVST group could be well labeled from cervical level (C)1 at E12.5 in the mouse and at d6 (HH stage 28) in the chicken. By contrast, we found that the cMVST group could not be well labeled from C1 until much later, but that we could label cMVST neurons as early as E13.5 in the mouse and d7.5 in the chicken, if tracer was applied in the MLF midway between cranial nerve nVIII and C1 (mid-medulla oblongata). To minimize sample variation within each species, we chose E13.5 in mice and d7.5 in chicken to retrogradely label both VS groups for manual cell isolation and RNA sequencing, applying tracer at C1 for the LVST, and the mid-medulla oblongata for the cMVST. For immunohistofluorescence, we always retrogradely labeled the LVST group from C1 regardless of stage, and we labeled the cMVST group from mid-medulla oblongata at E13.5 but from C1 at all later stages.

**Figure 1.**
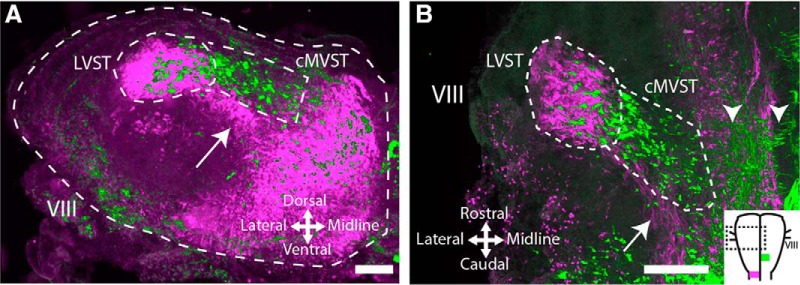
Spatial relationship between LVST and cMVST neuron groups. Differential retrograde labeling in the E13.5 mouse of LVST with RDA (pseudocolored magenta; applied unilaterally at C1), and cMVST with BDA/FDA (pseudocolored green; applied contralaterally at mid-medulla; ***B***, inset). Structures visible in the green channel have been superpositioned on structures visible in the magenta channel for clarity. ***A***, Overlay of 17 serial transverse sections spanning the rostrocaudal extent of the LVST/cMVST groups. Dotted white outlines show the section perimeter (outer) and the principal domains of the LVST and cMVST groups. LVST axons (arrow) course medioventrally to the LVST group. In this preparation, only 26 of 44 cMVST neurons that appear to be within the domain of the LVST group in the image projection are in fact intermingled with LVST neurons; the remainder actually lies outside the LVST group domain. ***B***, Overlay of nine frontal sections spanning the dorsoventral extent of the LVST/cMVST groups. LVST axons (arrow) course mediocaudally to the LVST group. Arrowheads indicate cMVST axons crossing the midline. In the inset, the dashed box shows the location of the full image, and magenta and green indicate dextran amine injection sites. VIII, Eighth cranial nerve entry site. Scale bar, 200 μm. See Figure 1-1, retrograde labeling from the mid-medulla does not label outside of the cMVST group.

Vestibulospinal neurons were retrogradely labeled for manual cell isolation with tetramethylrhodamine-conjugated dextran amine (RDA; 3 kDa; Invitrogen), or for immunohistofluorescence with a 1:1 mixture of fluorescein dextran amine (FDA; 3 kDa; Invitrogen) and biotin dextran amine (BDA; 3 kDa; Invitrogen) or pure BDA (Glover, 1995; Auclair et al., 1999). For labeling LVST neurons, a hemi-transection of the ventral half of the spinal cord was made at C1, and several small crystals of conjugated dextran amines were applied successively for at least 4 min to the transection. Preparations were then incubated in room temperature oxygenated ACSF or chicken ringer for at least 7 h for immunohistofluorescence, or at least 3 h for manual cell isolation. For labeling cMVST neurons, conjugated dextran amine crystals were applied to a hemi-transection of the brainstem, extending from the midline to ∼400 μm laterally, at the level midway between cranial nerve nVIII and C1. Tracer application at C1 is preferable, because it limits labeling to bona fide vestibulospinal axons, as opposed to axons that might terminate within the medulla. Thus, to control that this more rostral application did not label other neuron populations in the vicinity of the cMVST, we applied RDA unilaterally at C1, waited 6 h, and then applied FDA/BDA to the MLF on the same side at the mid-medullary level, in d11 chicken embryos (*n* = 2) and postnatal day (P)1 mice (*n* = 3). At these late stages most cMVST neurons have extended their axons to C1, and neurons labeled exclusively from mid-medulla could be quantified (FDA/BDA positive, RDA negative). In the chicken, 22 and 26%, and in the mouse, 14, 15, and 22% additional cells were labeled in the area of the cMVST from the mid-medullary compared with the C1 application. The additional cells for the most part were interspersed among the cMVST neurons labeled from C1 and appeared to be part of the same coherent neuron group (see Fig. 1-1), confirming that mid-medullary labeling does not lead to contamination of the cMVST group by non-cMVST neurons in that region.

### RNA sample acquisition

Retrograde labeling was performed with RDA as described above, with the addition of control lesions to minimize labeling of unwanted axonal pathways. The control lesions were performed within 10 min after RDA application, and only after complete removal of RDA from the preparation by focal superfusion followed by washing the preparation at least three times with ACSF or chicken ringer. After 3 h of incubation, tissue chunks restricted as closely as possible to the labeled LVST or cMVST groups were carefully dissected out, and placed in 1 mg/ml Pronase (Sigma-Aldrich) in ACSF or chicken ringer for 10 min at RT. Tissue was washed for 10 min in ACSF or chicken ringer, triturated to complete dissociation and dissociated RDA-labeled neurons manually sorted as described by Hempel et al. (2007). The isolated neurons were transferred to RNA extraction buffer (PicoPure RNA Isolation Kit; Applied Biosystems), heated for 30 min at 42°C, and stored at −80°C.

**Figure 2.**
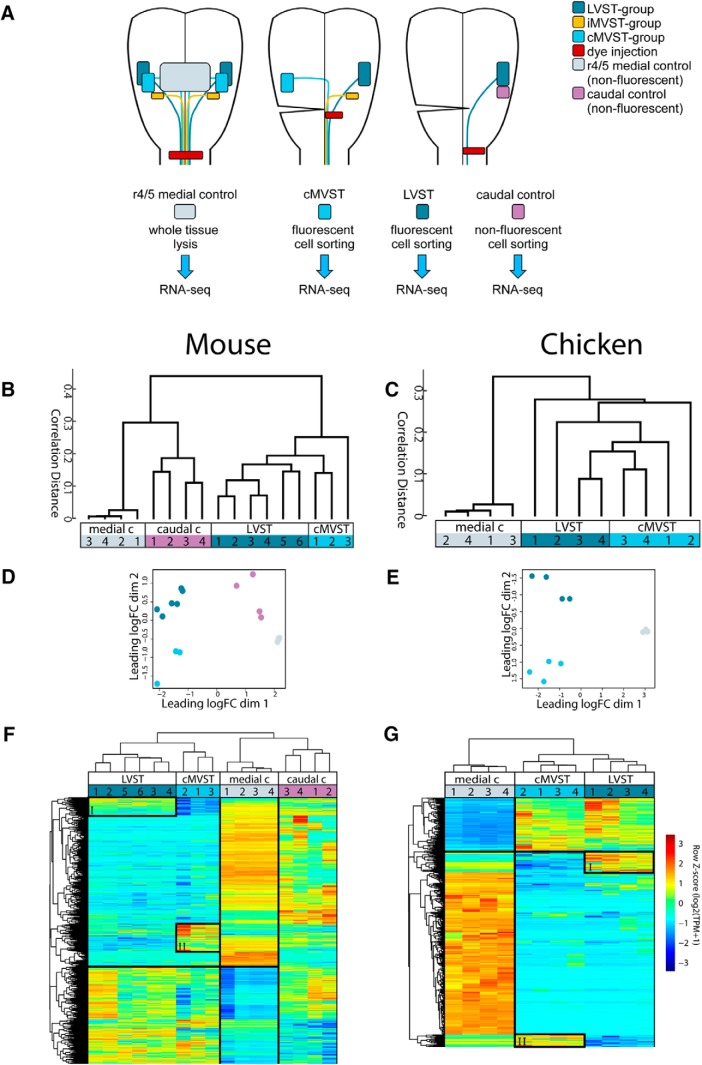
Mouse and chicken vestibulospinal neuron groups cluster separately based on whole-transcriptome gene expression profiles. ***A***, Schematic of E13.5 mouse or d7.5 chicken hindbrain showing anatomic locations of RDA injections, control lesions, and the retrogradely labeled VS neuron groups. LVST and cMVST group samples were dissociated and individual fluorescent cells sorted. Caudal control samples consisted of sorted nonfluorescent cells from a region immediately caudal to the LVST group (only collected in the mouse). Medial control samples consisted of tissue pieces at the same anteroposterior level as the VS neuron groups, laterally delimited by the retrogradely labeled LVST and cMVST groups. ***B***, ***C***, Hierarchical clustering plots showing correlation distance, and incorporating all genes that pass filter (based on cpm > 1 in at least 3 samples, one isoform per gene), the data are log2(TPM +1), distance metric is 1-Pearson correlation, linkage method is “ward”. ***D***, ***E***, Multidimensional scaling plots using the top 2000 highest variance genes (mouse; ***D***), or top 500 highest variance genes (chicken; ***E***), performed in edgeR. ***F***, ***G***, Biclustering of differentially expressed genes (FDR < 0.0005 ANOVA-like analysis and log2 fold-difference >1 between at least 2 sample groups) across samples in mouse (***F***) and chicken (***G***), performed in MATLAB using the clustergram function. Boxes labeled I and II highlight genes that are more abundant, respectively, in LVST than in cMVST samples, and vice versa. The thick black lines separate genes with an overall inverse expression pattern between the medial control and the vestibulospinal groups as a whole. Figure 2-1, RNA levels and fold-changes for all transcripts in LVST versus cMVST groups, normalized to control tissue. Figure 2-2, TPM values and ANOVA-like analysis for TFs from mouse RNAseq data. Figure 2-3, TPM values and ANOVA-like analysis for TFs from chicken RNAseq data.

We chose control tissue to include genes that would likely be commonly expressed at the same levels as the VS groups along the anteroposterior (AP) or the dorsoventral (DV) axis (note that in the hindbrain, the DV axis is anatomically displaced to the lateromedial axis, because of the dorsal opening of the fourth ventricle). Caudal control samples consisted of manually sorted nonfluorescent cells from a region (spanning 3–4 rhombomeres) immediately caudal to the LVST neuron group, and thus at a similar DV level. They were collected from the same preparations as the LVST neuron samples and contained only a few hundred cells each. Medial control samples were collected as bulk tissue from separate preparations, after bilateral RDA labeling from C1, with the sample extent delimited rostrocaudally and laterally by the locations of the RDA-labeled LVST and cMVST neuron groups, and thus derived from the same AP level (see Fig. 2*A*). Because they were bulk tissue samples, they contained many thousands of cells. The medial control samples were lysed directly after dissection, without trituration and dissociation of cells. In the mouse, we collected 6 LVST, 3 cMVST, 4 caudal control, and 4 medial control biological replicates. In the chicken, we collected 4 LVST, 4 cMVST, and 4 medial control biological replicates, but no caudal control samples. Each biological replicate was processed and sequenced independently, with ∼110 cells on average collected for each LVST and cMVST replicate. cMVST replicates each contained neurons pooled from at least three retrogradely labeled preparations, whereas each LVST replicate was obtained from a single preparation.

### RNA sequencing

mRNA was converted to cDNA and amplified with the Ovation RNA-seq System v2 kit (Nugen). Following amplification, cDNA was fragmented (∼250 bp) using a Covaris S2 sonicator, and ∼50 ng of fragmented cDNA was introduced into the Ovation Ultralow DR Multiplex System (Nugen) to generate bar-coded libraries. Quantification and quality control were assessed using Bioanalyzer 2100 (Agilent Technologies) and qPCR. Fifty base pair, single-end reads were generated on an Illumina HiSeq2500 platform for 2 of the mouse LVST samples and 2 of the caudal control samples, whereas 75 bp single-end reads were generated on an Illumina NextSeq500 for all other samples. Sequencing depth was minimum 19 million reads per sample. RNAseq data files have been uploaded to the Gene Expression Omnibus database (https://www.ncbi.nlm.nih.gov/geo/; Accession number GSE125197).

### RNAseq data analysis: mapping and alignment, dendrograms, clustergrams, MDS plots

**Figure 3.**
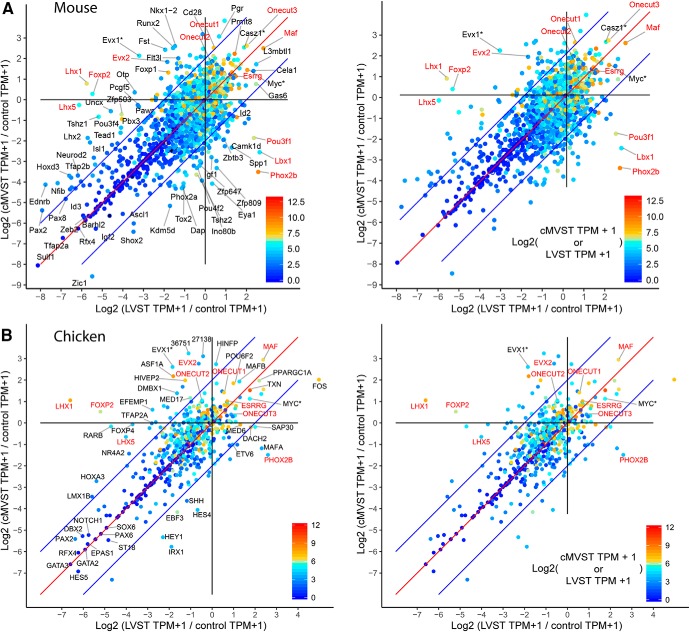
RNA levels and fold-change for TFs in LVST versus cMVST neurons, normalized to control tissue. Top row (***A***) shows mouse and bottom row (***B***) shows chicken data. ***A***, ***B***, Left, Names of genes with significant differential expression (FDR < 0.01, ANOVA-like analysis) and high fold-change between groups. Gene names of those TFs subsequently validated by immunohistofluorescence indicated in red (except for chicken ONECUT2, ONECUT3, and EVX2, for which antibodies were only functional in mouse). Right, Only the latter gene names. Chicken genes prefixed with ENSGALG and leading zeros have been shortened to non-zero digits only. Immunohistofluorescence for the TFs marked with an asterisk (Evx1*, Casz1*, and Myc*) stained most hindbrain cells above background level, and these genes were therefore not analyzed further. The *x*- and *y*-axes show, respectively, increasing levels of expression in the LVST and cMVST groups relative to control. The red diagonal indicates unity, and the color scale represents log2(TPM + 1) for cMVST group for points above the red diagonal, and for LVST group below the red diagonal, i.e., the VS group with the highest TPM value for each gene determines the point color. Genes above the upper blue diagonal have a >4-fold increase of TPM + 1 values for cMVST versus LVST group, and those below the lower blue diagonal have a >4-fold increase of TPM + 1 values for LVST versus cMVST group. For mouse, *n* = 2529 TFs; for chicken, *n* = 620 TFs. Figure 3-1, RNA level comparison of identified mouse/chicken TF orthologs.

Sequencing data in FASTQ format were aligned to the mouse genome (mm9) using the RUM pipeline v1.11 (Grant et al., 2011) or to the chicken genome (Galgal5) using the STAR aligner (Dobin et al., 2013). Genome feature quantification for mouse data were performed with RUM, using RefSeq annotations, and for chicken data with STAR using the Gallus_gallus.Gallus_gallus-5.0.93.gtf annotation file downloaded from Ensembl.org. Normalization of read counts, multidimensional scaling, and differential expression analyses were performed using edgeR (Robinson et al., 2010). Hierarchichal and biclustering were performed in MATLAB. Further details of analyses pertinent to figures is given in the figure legends. TFs were defined by Gene Ontology class 6355 (regulation of transcription, DNA templated). Identification of chicken-mouse TF orthologues (Fig. 3-1) was done in Ensembl Biomart (https://www.ensembl.org/biomart) using the “multi species comparisons” tool.

### Immunohistofluorescence

Retrogradely labeled brainstems were immersion fixed in 4% paraformaldehyde in PBS at 4°C for 30 min (embryonic preparations), or 1 h (d11 chicken, P1 mouse), then washed in PBS, sequentially incubated in 20% and 30% sucrose in PBS to equilibration, embedded in Tissue-Tek OCT embedding compound (Sakura), frozen in liquid nitrogen, and sectioned transversely at 14 μm using a cryostat. Sections were stored at −20°C or used directly. For immunohistofluorescence, sections were washed once in PBS for 5 min, once in 0.1% Tween 20 in tris-buffered saline (TBST) for 5 min, blocked in 10% normal donkey serum in TBST (blocking buffer) for 30 min at RT, incubated with primary antibodies in blocking buffer overnight at 4°C, and washed three times 5 min in PBS. They were then incubated with secondary antibodies, and/or fluorophore-conjugated streptavidins, and counterstained with 1 μg/ml Hoechst 33342 (Sigma-Aldrich) in TBST for 1 h at RT, washed three times 5 min in PBS, and mounted under coverslips in 1:1 PBS–glycerol or gelatin–H_2_O–glycerol 7 × *g*:42 ml:50 ml. Primary antibodies used are listed in Table 1. Secondary antibodies and fluorophore-conjugated streptavidins were obtained from Jackson Immuno Research and ThermoFisher Scientific, with secondary antibodies dilluted 1:1000 and streptavidin 1:500.

**Table 1.** Primary antibodies used

**Antigen**	**Immunogen**	**Host species**	**Reactivity**	**Source**	**Catalog#**	**RRID**	**Dillution**	**References**
Casz1^a^	Human Casz1 peptide	Rabbit	N/A	Rockland	600-401-B62S	AB_1961496	1:3000	
Esrrg	AA 2–100 of human Esrrg	Mouse	Mouse, chicken	R&D systems	PP-H6812-00	AB_2100280	1:1000	
Evx1^a^	AA 1–192	Rabbit	N/A	Dr. Martyn Goulding	N/A	N/A	1:300	(Moran-Rivard et al., 2001)
Evx2	AA 92–102 of mouse Evx2	Guinea pig	Mouse	Dr. Ryuichi Shirasaki	N/A	N/A	1:6 000	(Inamata and Shirasaki, 2014)
FoxP2	A peptide near N-terminus of human Foxp2	Goat	Mouse, chicken	Santa Cruz Biotechnology	sc-21069	AB_2107124	1:500	
Lbx1	Full-length mouse Lbx1	Guinea pig	Mouse, chicken	Dr. Thomas Müller	N/A	N/A	1:30,000	(Müller et al., 2002)
Lhx1/5	AA 1–360 of rat Lhx5	Mouse	Mouse, chicken	DSHB	4F2	AB_531784	1:15	(Tsuchida et al., 1994)
Maf	AA 150–200 of mouse Maf	Rabbit	Mouse, chicken	Bethyl Laboratories	A300-613	N/A	1:2000	
Myc^a^	AA 408–439 of human Myc	Mouse	N/A	DSHB	9E 10	AB_2266850	N/A	
Myc^a^	Full-length human Myc	Rabbit	N/A	Millipore	06-340	AB_310106	1:1000	
Myc^a^	AA ∼1–100 of human Myc	Rabbit	N/A	Abcam	ab32072 (Y69)	AB_731658	1:18,000	
Onecut1	Mix of AA 11–53 and 63–81 of mouse Onecut1	Guinea pig	Mouse	Dr. Frédéric Clotman	N/A	N/A	1:5000	(Espana and Clotman, 2012)
Onecut1	AA 11–110 of human Onecut1	Rabbit	Mouse, chicken	Santa Cruz Biotechnology	sc-13050	AB_2251852	1:300	
Onecut2	AA 185–326 of human Onecut2	Sheep	Mouse	R&D systems	AF6294	AB_10640365	1:500	
Onecut3	AA 23–333 of mouse Onecut3	Guinea pig	Mouse	Dr. Frédéric Clotman	N/A	N/A	1:6000	(Pierreux et al., 2004)
Phox2b	A peptide near N-terminus of human Phox2b	Goat	Mouse	Santa Cruz Biotechnology	sc-13224	AB_2251852	1:1000	
Phox2b	N/A	Rabbit	Chicken (mouse untested)	Dr. Jean-François Brunet	N/A	N/A	1:20,000	Unpublished
Pou3f1	A peptide near C-terminus of human Pou3f1	Goat	Mouse	Santa Cruz Biotechnology	sc-11661	AB_2268536	1:500	

a Antibodies that stained most cells in the hindbrain, or had excessive background signal. AA, Amino acids; DSHB, Developmental Studies Hybridoma Bank; N/A, not available.

### Imaging and quantification of TF expression

For assessment of colocalization and for quantification of TF expression in VS neurons, confocal *Z*-stacks of 2 μm optical sections were acquired from every other transverse section throughout the level of the VS groups (typically 10–15 sections) with Zeiss LMS510 meta, Zeiss LSM700, or Zeiss LSM710 microscopes, using N.A. 1.3 oil or N.A 1.2 water 40× objectives. Colocalization was determined manually, with care taken not to count VS neurons that lacked nuclear Hoechst staining (false-negatives), and avoiding false-positives arising from stacked cells in the *z*-axis (colocalization in *x*-*y* plane but not *z*-axis). This was facilitated by a customized ImageJ (Schneider et al., 2012) macro, which converted manually thresholded colocalized Hoechst and retrograde labeling signals to outlines, which were then superimposed onto contrast-enhanced immunostained images, from which positive and negative neurons for each TF could be counted.

Because of variability in their expression levels, Onecut1, 2 and 3 were quantified in the mouse LVST group both in terms of numbers of neurons and immunostaining intensity. This was done by measuring fluorescent signal intensity (average pixel value) in every retrogradely labeled VS neuron in every fourth transverse section per embryo. Positive versus negative immunostaining was determined using a per embryo threshold value, setting a value that distinguished two distinct populations, one corresponding to negative or very weakly stained neurons, the other more intensely stained neurons.

Counts and *x*-*y*-*z* coordinates of all neurons immunostained for a given TF were obtained from at least three embryos per TF at each stage. Coordinates were plotted as 2D scatterplots and min–max normalized histograms for each cardinal axis using MATLAB Release 2015b (MathWorks).

### CNS-wide triple and quadruple TF colocalization analysis, and 3D reconstruction

To determine the location and uniqueness of TF signatures, large-scale 3D reconstructions covering the entire brainstem, and (where necessary) the cerebrum and portions of the spinal cord, were generated from images acquired with a Axio Scan Z1 microscope (Zeiss), or a Panoramic Midi microscope (3D Histech) with N.A. 0.8 air 20× objectives. Colocalization for each combination of TFs was evaluated in 14 μm immunostained cryosections, in at least three individual embryos. Inspection and counting was done in either the left or the right half of at least every sixth transverse section of the brainstem, and where necessary, every sixth parasagittal section of the cerebrum, and at least three transverse sections each from the lumbar, cervical, and thoracic spinal cord. Identification of neurons with triple- or quadruple-colocalized TFs was done by thresholding individual imaging channels either manually or using the built-in automatic local threshold plugin in ImageJ. The resulting masks were applied to successive AND operations with the ImageJ image calculator until a single mask of potential colocalized TF pixels was generated. This mask was used as an aid in locating colocalized TFs, but care was taken to verify the validity of each thresholded channel against the original image, and manual inspection was employed where thresholding failed or was uncertain. 3D scatterplots with hindbrain outlines were generated using ImageJ to extract coordinates of colocalized staining and tissue contours from transverse sections. These were then plotted using MATLAB Release 2015b (MathWorks), with the alphashape and 3D scatterplot functions. For quantifying colocalization of retrogradely labeled LVST neurons and Phox2b/Esrrg/Maf, or r5-cMVST neurons and Evx2/Esrrg/Maf, only Phox2b- (LVST marker) or Evx2- (r5-cMVST marker) positive neurons were considered, to avoid counting neurons that lacked nuclei (false-negatives).

## Statistical table

Statistical analyses were conducted in the R software v3.3.3 (https://www.r-project.org/) and MATLAB Release 2015b (MathWorks). Two-sample Kolmogorov–Smirnov tests were calculated with the MATLAB function kstest2. See the RNAseq data analysis paragraph in Materials and Methods for additional details on RNAseq data analysis (Table 2).

**Table 2.** Statistical table

**Data structure**	**Parameter tested**	**Type of test**	***P***	**Figure**
RNAseq data passing filter (counts per million > 1 in at least 3 samples), grouped by sample type	Gene counts	Gene by gene ANOVA-like differential abundance analysis to test for differences between any sample groups using edgeR	FDR < 0.1	Used as filter criteria in 3, and 2-1*A*,*B*. Specific values listed in 2-2, 2-3. For 2*F*,*G* a more stringent threshold of FDR < 5e−4 was used.
Two independent samples: r5 and r4 cMVST cell population, pooled normalized data from *n* = 3 animals.	Esrrg fluorescence intensity	Two-sample Kolmogorov–Smirnov test	3.6e-7	7*C*

## Results

### The LVST and cMVST are hodologically distinct neuron groups with limited spatial overlap

We first characterized the spatial relationships of the LVST and cMVST neuron groups by retrograde labeling and confocal microscopy. Differential retrograde labeling of the LVST and cMVST with conjugated dextran amines in the mouse embryo never led to double-labeled neurons (Fig. 1), demonstrating that the LVST and cMVST neuron groups are hodologically distinct, as previously demonstrated in the chicken embryo (Díaz et al., 2003). However, there was some spatial overlap, in which ∼ 20% of cMVST neurons were located within the domain of the LVST group (Fig. 1). This highlights the need for an approach employing retrograde labeling and cell sorting to purify these neuron groups before transcriptomic characterization.

10.1523/ENEURO.0475-18.2019.f1-1 Figure 1-1 Retrograde labeling from mid-medulla does not label outside of the cMVST group. cMVST group in the d11 chicken embryo retrogradely labeled from C1 (***A***), and mid-medulla (***B***). cMVST group in the P1 mouse retrogradely labeled from C1 (***C***), and mid-medulla (***D***). Scale bar, 200 μm. Download Figure 1-1, TIF file.

### Global RNAseq profiles of LVST and cMVST neuron groups reveal differential transcript abundance across numerous genes, including those encoding TFs

Having determined the appropriate early stages for manual cell sorting following retrograde labeling (mouse E13.5, chicken d7.5; see Materials and Methods) we performed RNAseq on samples from the LVST group, the cMVST group, and control tissue located medially or caudally to the VS groups (Fig. 2A). We selected these control samples to distinguish VS-enriched transcripts from those commonly expressed at similar AP or DV locations (Fig. 2*A*). Unsupervised clustering algorithms, blinded to labeling strategy, generated groupings that ultimately aligned with labeled hodological identity or regional origin (caudal control and medial control; Fig. 2*B*–*E*). Differential expression analysis identified significantly differentially expressed transcripts across retrogradely labeled neuron and control sample groups (Figs. 2*F*,*G*, 3, and Figs. 2-1, 2-2, 2-3).

10.1523/ENEURO.0475-18.2019.f2-1 Figure 2-1 RNA levels and fold-changes for all transcripts in LVST versus cMVST, normalized to control tissue. All detected transcripts in mouse (***A***) and chicken (***B***) from RNAseq data (13,844 mouse and 11,982 chicken genes). ***A***, ***B***, Legend as in Figure 3. ***C***, Transcript levels of select highly differentially expressed non-TF genes in individual mouse RNAseq samples. ***D***, Transcript levels of same genes as in ***C*** for individual chicken RNAseq samples. Download Figure 2-1, TIF file.

10.1523/ENEURO.0475-18.2019.f2-2 Figure 2-2 TPM values and ANOVA-like analysis for TFs from mouse RNAseq data. Values calculated with ANOVA-like differential abundance analysis performed in in edgeR. TF inclusion criteria based Gene Ontology class 6355 (regulation of transcription, DNA templated) and counts per million >1 in at least three samples. Columns: (***A***) assigned gene index, (***B***) gene name, (***C***) RefSeq ID, (***D***) transcript length in nucleotides, (***E***) likelihood ratio statistics, (***F***) two-sided *p* value, (***G***) false discovery rate adjusted *p* value, (***H***) LVST mean TPM value, (***I***) cMVST mean TPM value, (***J***) mean caudal control sample TPM value, (***K***) mean medial control sample TPM value, (***L***–***Q***) individual LVST sample TPM values, (***R***–***T***) individual cMVST sample TPM values, (***U***–***X***) individual caudal control sample TPM values, (***Y***–***AB***) individual medial control sample TPM values. Download Figure 2-2, XLSX file.

10.1523/ENEURO.0475-18.2019.f2-3 Figure 2-3 TPM values and ANOVA-like analysis for TFs from chicken RNAseq data. Values calculated with ANOVA-like differential abundance analysis performed in in edgeR. TF inclusion criteria based Gene Ontology class 6355 (regulation of transcription, DNA templated) and counts per million >1 in at least three samples. Columns legend: (***A***) assigned gene index, (***B***) gene name, (***C***) ensembl ID, (***D***) transcript length in nucleotides, (***E***) likelihood ratio statistics, (***F***) two-sided *p* value, (***G***) false discovery rate adjusted *p* value, (***H***) LVST mean TPM value, (***I***) cMVST mean TPM value, (***J***) mean medial control sample TPM value, (***K***–***N***) individual LVST sample TPM values, (***O***–***R***) individual cMVST sample TPM values, (***S***–***V***) individual medial control sample TPM values. Download Figure 2-3, XLSX file.

10.1523/ENEURO.0475-18.2019.f3-1 Figure 3-1 RNA level comparison of identified mouse/chicken TF orthologs. Plots show identified TFs that shared orthologues in mouse and chicken, plotted for Log2(TPM + 1) values, with mouse values on the *x*-axis and chicken values on the *y*-axis, for the LVST groups (***A***) and cMVST groups (***B***). Mouse-distinct TFs are defined as having Log2(TPM + 1) value >4 in mouse, and <1 in chicken, and vice versa. TF gene names (only mouse names used) shown in red indicate TFs whose expression was validated by immunohistofluorescence. Diagonal line shows the linear regression line with slope, intercept and r2 values shown at the top left in each plot. Download Figure 3-1, TIF file.

Clustergrams of differentially expressed genes highlight several inverse patterns between groups (Fig. 2*F*,*G*). The vestibulospinal groups as a whole show an inverse pattern compared with the medial control, as expected from their different dorsolateral origins within the same rhombomeres (r4–r5). The relationship to the caudal control is more complex, likely because the caudal control covered multiple (3–4) rhombomeres and also comprised far fewer cells (manually collected) than the medial control (bulk tissue). Most importantly, some genes, including those encoding particular TFs, show an inverse pattern between the LVST and cMVST groups, being exclusive to or enriched in one or the other (Figs. 2*F*,*G*, 3, and Figs. 2-1, 2-2, 2-3).

A primary goal of our study was to identify candidate genes that might function as key regulators of VS neuron subtype identity, thus our main focus in further analysis of the LVST and cMVST neuron group transcriptomes was on TF-encoding genes. Nevertheless, we noted that several non-TF genes were highly differentially expressed between the LVST and cMVST neuron groups or between these and controls (Fig. 2-1). In the mouse, the LVST group samples showed higher levels of *Islr2* (Linx; Ig superfamily containing leucine rich repeat 2), *Cdh22* (cadherin 22), *Cbln1* and *Cbln4* (cerebellin 1, and 4), and *Calb2* (calretinin) transcripts compared with the cMVST group samples, whereas the cMVST group samples showed greater abundance of *Sst* (*Somatostatin*), *Sema5a* (*Semaphorin 5a*), and *Slc32a1* (*Vgat*) RNAs. Examples of transcripts enriched in both VS groups compared with control samples were *Tll1* (Tolloid like 1; metalloprotease), *Ntng2* (Netrin G2), *Rbp1* (retinol binding protein 1), and *Cryba2* (crystallin beta A2; Eye Lens Structural Protein; Fig. 2-1*C*). Some of these showed similar relative differences in transcript levels in chicken (Fig. 2-1*D*).

### Conserved expression of common and exclusive VS group TFs

To identify candidate TFs for further investigation, we considered RNA abundance against control tissue (Fig. 3, and Figs. 2-2, 2-3), the absolute level of each particular TF-encoding transcript (Fig. 3, and Figs. 2-2, 2-3), the degree to which group-specific expression in mouse and chicken were conserved (Figs. 2-2, 2-3, 3-1), and by consulting the literature. Lhx1, Lhx5, Foxp2, Evx2, Onecut1, Onecut2, Onecut3, Esrrg, Maf, Phox2b, Lbx1, and Pou3f1 were all selected for further analysis by immunohistofluorescence, based on these criteria and the availability of functional antibodies. Evx1, Casz1, and Myc were also considered, but available antibodies stained most neurons in the hindbrain at embryonic stages, or had excessive background signal, and thus were not pursued further. Available antibodies against Evx2, Onecut2, Onecut3, and Pou3f1 were not functional in chicken embryonic tissue.

**Figure 4.**
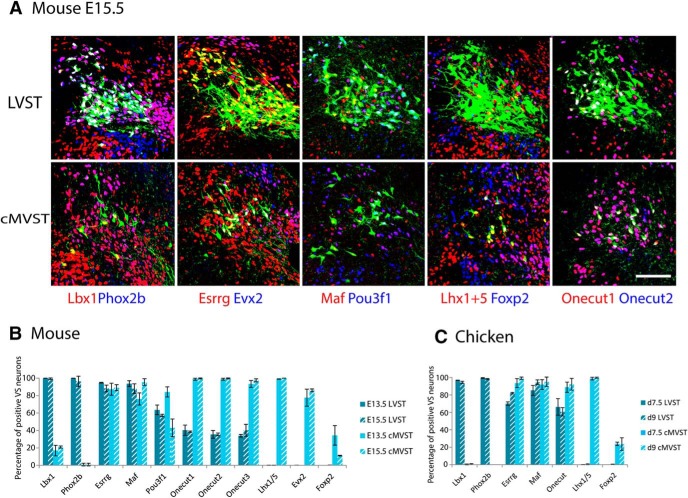
Immunohistofluorescent staining for TFs in LVST and cMVST neurons. ***A***, Confocal images of transverse sections through the level of the VS neurons in an E15.5 mouse. Retrogradely labeled LVST neurons (top row) and cMVST neurons (bottom row) shown in green, and immunostaining for TFs in colors as indicated below each column. Overlapping LVST and Lhx1+5 pixels (yellow) in the LVST/Lhx1+5 panel do not colocalize in the *z*-axis, and are thus false-positives (see Materials and Methods). Scale bar, 100 µm. Dorsal, up; lateral, left. ***B***, ***C***, Percentage of retrogradely labeled LVST or cMVST neurons immunopositive for the indicated TFs in E13.5 and E15.5 mouse embryos (***B***) and d7.5 and d9 chicken embryos (***C***). Error bars represent SD. Figure 4-1, Histograms and scatterplots showing the spatial distribution of mouse LVST neurons expressing the indicated TFs. Figure 4-2, Histograms and scatterplots showing the spatial distribution of chicken LVST neurons expressing the indicated TFs. Figure 4-3: Histograms and scatterplots showing the spatial distribution of mouse cMVST neurons expressing the indicated TFs. Figure 4-4, Histograms and scatterplots showing the spatial distribution of chicken cMVST neurons expressing the indicated TFs. Figure 4-5, Percentage of mouse LVST neurons immunopositive for a single Onecut transcription factor. Figure 4-6, Differential Onecut TF staining intensity in the cMVST neuron group at different rostro-caudal levels.

Immunostaining revealed TFs that were expressed at the protein level in the VS groups at the stages we examined (E13.5 and E15 in mouse embryo, and d7.5 and d9 in chicken embryo). Qualitative assessment of TF expression showed that LVST neurons were immunopositive for Phox2b, Lbx1, Esrrg and Maf, immunonegative for Lhx1+5, Evx2 and Foxp2, and that subpopulations were immunopositive for Onecut1/2/3 and Pou3f1 (Fig. 4*A*). By contrast, cMVST neurons were immunopositive for Lhx1+5, Esrrg, Maf and Onecut1/2/3, immunonegative for Phox2b, and subpopulations were immunopositive for Lbx1 (mouse only), Pou3f1, Evx2, and Foxp2 (Fig. 4*A*). Detailed quantification revealed consistency in this pattern across stages and species, but the absence of an Lbx1+ domain in the chicken cMVST group, and fewer Esrrg+ cells in chicken versus mouse LVST group, were notable exceptions (Fig. 4*B*,*C*).

10.1523/ENEURO.0475-18.2019.f4-1 Figure 4-1 Histograms and scatterplots showing the spatial distribution of mouse LVST neurons expressing the indicated TFs. Neurons are indicated as either immunopositive (blue) or immunonegative (red). Histograms show averages and SD along the indicated axes. Scatterplots show projections in the indicated planes from single, representative preparations for each TF and stage. Download Figure 4-1, TIF file.

10.1523/ENEURO.0475-18.2019.f4-2 Figure 4-2 Histograms and scatterplots showing the spatial distribution of chicken LVST neurons expressing the indicated TFs. Legend as Figure 4-1. Download Figure 4-2, TIF file.

10.1523/ENEURO.0475-18.2019.f4-3 Figure 4-3 Histograms and scatterplots showing the spatial distribution of mouse cMVST neurons expressing the indicated TFs. Legend as Figure 4-1. Download Figure 4-3, TIF file.

10.1523/ENEURO.0475-18.2019.f4-4 Figure 4-4 Histograms and scatterplots showing the spatial distribution of chicken cMVST neurons expressing the indicated TFs. Legend as Figure 4-1. Download Figure 4-4, TIF file.

**Figure 5.**
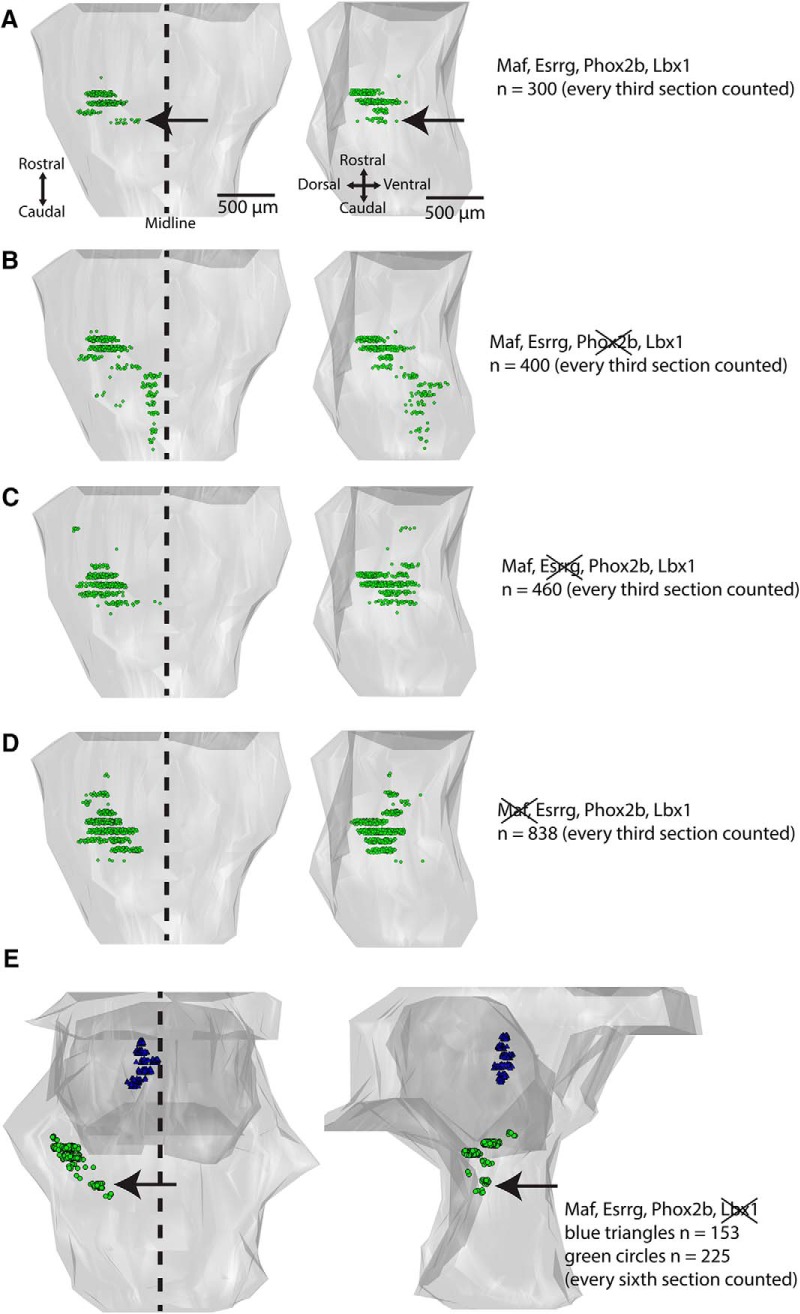
Spatial restriction of the Maf/Esrrg/Phox2b/Lbx1 LVST neuron group signature compared with different three-way combinations thereof. ***A***–***D***, Each panel shows a 3D reconstruction of the E13.5 mouse hindbrain with the neurons (*n*) coexpressing the indicated set of TFs shown as green circles. Neurons are plotted from every third serial transverse section. ***A***, Most neurons expressing Maf/Esrrg/Phox2b/Lbx1 lie within the LVST neuron group domain (by comparison with adjacent serial sections with retrograde labeling), but a few lie in a more medioventral domain (arrow indicates this smaller, mediolateral vicinal neuron group). ***B***–***D***, Neurons expressing the indicated 3-TF signatures are found in the LVST group domain and in additional domains within the hindbrain. ***E***, 3D reconstruction of the E13.5 mouse hindbrain and midbrain, with neurons coexpressing Maf, Esrrg, and Phox2b indicated as green circles within the hindbrain and blue triangles within the midbrain. Neurons expressing this 3-TF signature were found in the domains of the LVST group, the smaller vicinal mediocaudal neuron group (arrow) and a neuron group in the midbrain near the midline (blue triangles). Neurons are plotted from every sixth serial transverse section.

10.1523/ENEURO.0475-18.2019.f4-5 Figure 4-5 Percentage of mouse LVST neurons immunopositive for a single Onecut TF. Percentage ± SD of mouse LVST neurons at indicated stages that were singly immunopositive for one (of two) Onecut factors. Oc, Onecut. Download Figure 4-5, DOCX file.

**Figure 6.**
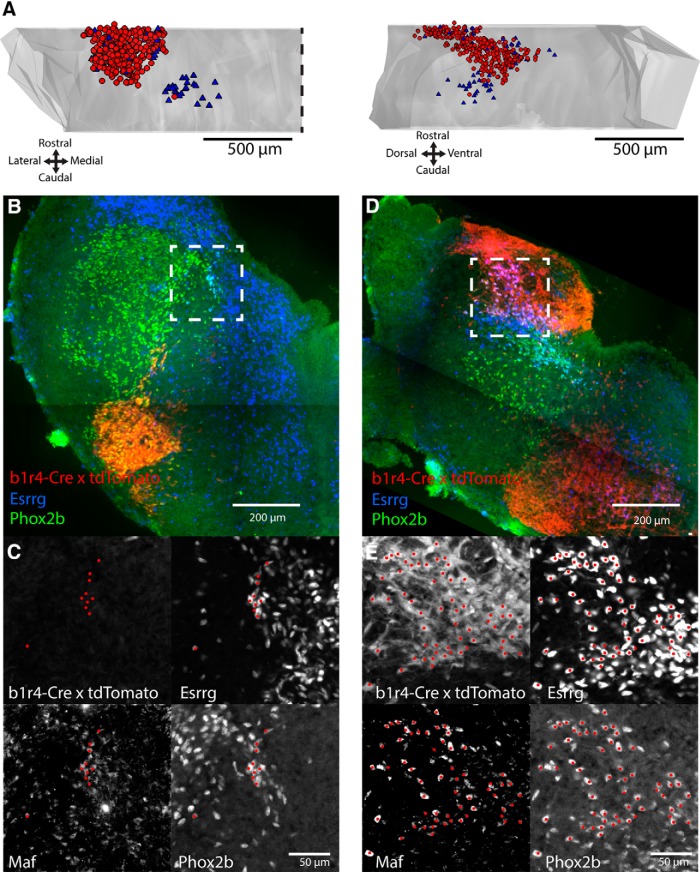
The LVST neuron group and the vicinal mediocaudal neuron group sharing the LVST group TF signature derive from different rhombomeric lineages. ***A***, Expression of Maf/Esrrg/Phox2b confirmed in LVST neurons by direct combination with retrograde BDA labeling in the E13.5 mouse hindbrain. Maf/Esrrg/Phox2b triple-positive neurons were restricted to two clusters, the large LVST neuron group and the smaller, vicinal mediocaudal neuron group, seen in frontal (left) and lateral (right) views. Red circles, Maf+ Esrrg+ Phox2b+ BDA+ neurons (*n* = 306); blue triangles, Maf+ Esrrg+ Phox2b+ BDA- neurons (*n* = 59). Cells from every third section in this preparation counted. Dashed line indicates the midline. ***B***–***E***, Transverse sections from an E14.5 b1r4-Cre x tdTomato mouse, triple-immunostained for Maf, Esrrg, and Phox2b, at the level of the vicinal mediocaudal neuron group (***B***, ***C***) and the LVST neuron group (***D***, ***E***). ***C***, ***E***, Separate confocal images of each fluorescent channel from within the dashed boxes shown in ***B*** and ***D***, with red dots indicating cells colabeled for Maf/Esrrg/Phox2b. tdTomato staining is absent in the vicinal mediocaudal neuron group (***C***), indicating the origin of these neurons from outside of r4. The anti-Maf signal is not shown in ***B*** or ***D*** for clarity. ***B***–***E***, Dorsal, up; lateral, left; midline, right.

10.1523/ENEURO.0475-18.2019.f4-6 Figure 4-6 Differential Onecut TF staining intensity in the cMVST neuron group at different rostrocaudal levels. ***A***–***L***, Confocal images of transverse sections through the E13.5 mouse cMVST, retrogradely labeled with BDA from the mid-medulla. Immunostained with antibodies specific for the indicated Onecut TFs. Imaging settings and contrast levels preserved between different rostrocaudal levels. Red dots in ***A***–***C***,***G***–***I*** indicate the locations of corresponding cMVST neurons in ***D***–***F***,***J***–***M***. ***A***–***F***, The mid rostrocaudal level of the cMVST group; ***G***–***L***, the rostral level of the cMVST group. Immunostaining for Onecut TFs is stronger in rostral cMVST neurons. Dorsal, up; lateral, left; medial, right. Scale bar, 50 µm. Oc, Onecut. Download Figure 4-6, TIF file.

Although most of the cell-type-specific immunostaining patterns we examined correlated with the transcript-level differences identified by RNAseq (Table 3), one exception was Lbx1 expression. We did not detect *Lbx1* mRNA by RNAseq in the mouse cMVST group, or in any of the chicken samples (Table 3), despite positive immunostaining for Lbx1 protein in a subpopulation of the mouse cMVST neurons (Fig. 4*A*,*B*), and in nearly all of the chicken LVST neurons (Fig. 4*C*). Interestingly, mouse *Lbx1* transcript levels were an order of magnitude lower in the LVST group [36 ± 30 transcripts per million (TPM)], compared with Phox2b (678 ± 126 TPM), Maf (495 ± 165 TPM), and Esrrg (753 ± 124 TPM), despite all of these TFs being positively immunostained in nearly all LVST neurons. Thus low transcript abundance, potentially outside the range of sensitivity of our RNA-seq approach, may nonetheless be sufficient for translation of the encoded protein as demonstrated in the case of *Lbx1* in some of our sorted neuron pools.

**Table 3.** Immunostaining patterns and RNA levels of TFs in vestibulospinal neurons

**Gene**	**Species**	**Groups immunostained**	**LVST TPM**	**cMVST TPM**
Phox2b	Mouse	LVST	678 ± 126	9 ± 3
	Chicken	LVST	16 ± 20	0 ± 0
Lbx1	Mouse	LVST + cMVST	36 ± 30	0
	Chicken	LVST	0 ± 0	0 ± 0
Maf	Mouse	LVST + cMVST	495 ± 165	390 ± 125
	Chicken	LVST + cMVST	55 ± 35	82 ± 23
Esrrg	Mouse	LVST + cMVST	753 ± 124	588 ± 272
	Chicken	LVST + cMVST	143 ± 106	177 ± 85
Pou3f1	Mouse	LVST + cMVST	103 ± 41	5 ± 2
	Chicken	N/A	0 ± 0	0 ± 0
Onecut1	Mouse	LVST + cMVST	40 ± 23	175 ± 75
	Chicken	LVST + cMVST	50 ± 28	92 ± 49
Onecut2	Mouse	LVST + cMVST	112 ± 20	275 ± 128
	Chicken	N/A	2 ± 3	8 ± 6
Onecut3	Mouse	LVST + cMVST	87 ± 61	131 ± 99
	Chicken	N/A	10 ± 10	6 ± 5
Lhx1	Mouse	cMVST	0	115 ± 35
	Chicken	cMVST	0 ± 0	200 ± 121
Lhx5	Mouse	cMVST	0	57 ± 19
	Chicken	cMVST	1 ± 2	18 ± 14
Foxp2	Mouse	cMVST	0	59 ± 27
	Chicken	cMVST	0 ± 0	65 ± 15
Evx2	Mouse	cMVST	0	12 ± 10
	Chicken	N/A	0 ± 0	9 ± 8

Mean TPM values (±SD) of TFs validated by immunohistofluorescence, showing a strong correlation of VS group RNA levels and immunostaining. A value of 0 indicates no detection by RNAseq. N/A, Not assessed by immunohistofluorescence because of lack of appropriate antibodies.

### A restricted, conserved TF signature that uniquely defines the LVST group within the r4 lineage

Immunostaining with the TF panel obtained for the LVST group showed that some TFs were expressed in all LVST neurons, whereas some were expressed in only part of the LVST group. In the mouse, Phox2b and Lbx1 were expressed in virtually all LVST neurons, Esrrg and Maf were expressed in 90%-95% of LVST neurons, whereas Onecut 1, 2, and 3 and Pou3f1 were expressed in smaller subpopulations of LVST neurons (Fig. 4*A*,*B*). Results were similar in chicken, with Phox2b and Lbx1 expressed in virtually all LVST neurons and Maf in 85–95% of LVST neurons. However, Esrrg was expressed in only ∼ 70–80% of LVST neurons, contrasting the 90–95% observed in mouse.

#### Spatial restriction of the 4-TF signature

Noting that 4 TFs (Phox2b/Lbx1/Maf/Esrrg) were expressed in most of the LVST neurons, we then asked how restricted is the expression of this signature in the developing CNS. We assessed this with quadruple combinatorial immunostaining in the E13.5 mouse. Because Phox2b, and consequently the 4-TF combination, is not expressed outside of the region spanning from the midbrain through the thoracic spinal cord (Pattyn et al., 1997), we first confirmed the cessation of Phox2b immunostaining at these levels and then limited our analysis to that range. We found that cells expressing the 4-TF combination were restricted to a short stretch of the dorsolateral hindbrain. Being limited by our available fluorophore and microscope capacity to simultaneously visualize four fluorophores in any given section, we stained for retrogradely labeled LVST neurons in alternating sections. This indicated that the 4-TF combination was restricted to the LVST group, plus a smaller domain just mediocaudal to the LVST group (Fig. 5*A*).

#### The 4-TF signature cannot be reduced to a 3-TF signature

To determine whether any 3-TF combinations among the 4 had similar spatial restriction, we assessed each separately in serial sections spanning the midbrain, hindbrain, and cervical and thoracic spinal cord. This showed that within the hindbrain, only one 3-TF combination (Maf/Esrrg/Phox2b) had the same spatial restriction as the 4-TF combination (Fig. 5, compare *A*, *E*). Other 3-TF combinations were each expressed by additional specific outlying neuron groups within the hindbrain (Fig. 5*B*–*D*). However, the Maf/Esrrg/Phox2b combination was also expressed by a group of neurons lying medially within the midbrain at the rostral end of the Phox2b CNS domain (Fig. 5*E*). Thus, only the 4-TF signature Maf/Esrrg/Phox2b/Lbx1 appeared to be expressed exclusively by the LVST group and its associated small vicinal mediocaudal group.

#### Use of the 3-TF signature as a proxy for the 4-TF signature within the hindbrain

The equivalence within the hindbrain (excluding the midbrain) of the immunostaining pattern for the 3-TF combination Maf/Esrrg/Phox2b and the 4-TF signature allowed us to use the former as a proxy for the latter in subsequent experiments. We could then directly assess the expression of the 3-TF combination in serial sections in which the LVST group was also retrogradely labeled, instead of indirectly in adjacent sections as done above. This showed unequivocally that the 4-TF signature was restricted to the LVST group and the smaller vicinal mediocaudal neuron group at E13.5 (Fig. 6*A*). We repeated the experiment in the E15.5 mouse hindbrain with identical results (Table 4). At E13.5, 86 and 90% of LVST neurons expressed this 4-TF combination in the two preparations assessed in this way (the remaining minority lacking either Maf or Esrrg; compare Fig. 4*B*).

**Table 4.** Developmental appearance of the 3-TF proxy for the LVST neuron group signature

**Stage**	**Mediocaudal vicinal group**	**LVST neuron group**	**Midbrain group**	**N preparations**	**Sections assessed per preparation**
	**(Lbx1+, Hoxb1-)**	**(Lbx1+, Hoxb1+)**	**(Lbx1-, Hoxb1-)**		
E9.5	0	0	0	3	Every 4th
E11.5	0, 0	152, 59	N/A	2	Every 4th
E13.5	31 ± 4	203 ± 41	145 ± 13	3	Every 6th
E15.5	31 ± 13	186 ± 66	N/A	3	Every 6th

Mean (±SD) of Maf/Esrrg/Phox2b triple-positive hindbrain neurons at different locations (with indicated coexpression of either Lbx1 and/or Hoxb1) at different developmental stages in the mouse embryo. N/A, Not assessed.

#### The 4-TF signature is unique to the LVST within the r4 lineage

We have not yet determined the identity of the vicinal mediocaudal neuron group, except to confirm that it was not retrogradely labeled from the spinal cord (Fig. 6*A*), nor have we identified alternative TF signatures that differentiate it from the LVST group. We noted, however, that a consistent feature of this neuron group was its relatively weak Maf immunostaining compared with the LVST group (Fig. 6*C*,*E*). Its more caudal location also prompted us to assess whether the two groups might have different rhombomeric origins. To do this, we used *b1r4-Cre x tdTomato* mice (Di Bonito et al., 2013) in which Cre expression and activity is restricted to r4 by a *HoxB1* r4-enhancer element, and tdTomato expression is thereby restricted to cells descendant from r4 (see Methods and Materials). In these mice, we found that of the two neuron groups that expressed the 3-TF combination, only the LVST group derived from r4-limited *HoxB1-*expressing progenitors at E14.5 (Fig. 6*B*–*E*). Thus, the 4-TF signature Maf/Esrrg/Phox2b/Lbx1 uniquely defines the LVST group within the r4 lineage.

#### The 4-TF signature emerges at an early stage

Having identified a lineage-specific TF signature that uniquely defines the LVST neuron group, we then asked how early during mouse hindbrain development this TF signature arises (Table 4). At E9.5, Phox2b immunostaining was evident in progenitors in the ventricular zone, but there were virtually no Maf, Esrrg or Lbx1 immunopositive cells in the hindbrain. At E11.5, we observed triple-positive Maf/Esrrg/Phox2b neurons (*n* = 2 preparations) and Esrrg/Phox2b/Lbx1 neurons (*n* = 2 preparations) overlapping with retrograde labeling of the nascent LVST group. We found that the Maf/Esrrg/Phox2b triple-positive neurons clustered in a single group in the hindbrain, and 20–50% of these were retrogradely labeled. E11.5 is during the period when LVST axons are growing toward the spinal cord, so some LVST neurons are expected to not be retrogradely labeled because their axons have not yet reached the tracer application site at C1. The percentage of Maf/Esrrg/Phox2b triple-positive neurons within the presumptive LVST domain that were retrogradely labeled from C1 increased to ∼70 and 95% at E13.5 and E15.5, respectively.

### TFs that define LVST neuron subpopulations

Next, we asked whether any of the TFs that were expressed by only a fraction of the LVST group were spatially compartmentalized within the group. We did this by generating 3D reconstructions of the immunostained LVST neurons (Figs. 4-1, 4-2). The examples described below of TFs with differential regional expression suggest a partitioning of LVST neurons into anatomic or functional subpopulations.

#### Esrrg in chicken

As already noted, in the mouse, virtually all LVST neurons expressed Esrrg. By contrast, in the chicken, Esrrg was only expressed by 70–80% of LVST neurons. 3D reconstructions showed that Esrrg-negative neurons in the chicken formed a coherent subpopulation located dorsolaterally within the LVST group (Fig. 4-2).

#### Pou3f1 in mouse

Approximately 60% of LVST neurons were Pou3f1+ in the mouse. At E13.5 in the mouse there was no obvious spatial pattern in the Pou3f1 expression pattern: Pou3f1+ and Pou3f1-negative LVST neurons were intermingled. By E15.5, however, the Pou3f1+ subpopulation occupied the dorsolateral, rostral part of the LVST (Fig. 4-1). In the chicken, *POU3F1* is listed as a pseudogene in the Gallus_gallus.Gallus_gallus-5.0.93.gtf annotation file downloaded from Ensembl. In accordance with this we could not detect POU3F1 by immunohistofluorescence (nor by RNAseq).

#### Onecut TFs

Similar proportions (∼40%) of mouse LVST neurons expressed Onecut1, 2, and 3, and these were all located more ventrocaudally within the LVST (Fig. 4-1). In the chicken, for which we had antibodies only against Onecut1, the picture was similar: ∼60% of LVST neurons expressed Onecut1, and these were located more caudally within the LVST (Fig. 4-2).

By immunostaining for Onecut1+2 or Onecut2+3 concurrently, we determined that only a minority of LVST neurons in the mouse (∼5%) was single-positive for one of the two Onecut factors (Fig. 4-5). From this we deduced that most LVST neurons express either all Onecut factors, or none of them. Onecuts 1, 2, and 3 displayed a wide range of immunostaining intensities in mouse LVST neurons. By measuring this within individual LVST neurons we determined that Onecut2 and Onecut3 immunostaining intensity correlated highly (E13.5: *R* ^2^ = 0.93 ± 0.01, E15.5: *R* ^2^ = 0.88 ± 0.06), whereas Onecut1 and 2 correlated less (E13.5: *R* ^2^ = 0.56 ± 0.14, E15.5: *R* ^2^ = 0.52 ± 0.14). A characteristic feature of the mouse LVST group at E15.5 was a rostral and very dorsal subpopulation of LVST neurons that consistently included only a few Onecut1/2/3+ neurons, and essentially no Esrrg-negative or Pou3f1-negative neurons (Fig. 4-1).

### TF combinations that distinguish r4- versus r5-derived cMVST neurons

In contrast to the LVST group, which derives exclusively from r4, the cMVST group derives from both r4 and r5, with the major part derived from r5. Our initial assessment of TF transcript expression indicated that virtually all cMVST neurons in the mouse express Lhx1+5 and Onecut 1, 2, and 3 at both E13.5 and E15.5 (Fig. 4*A*,*B*). However, Onecut3 stained most cMVST neurons only very weakly, except in the rostral portion of the cMVST group, where all Onecut factors exhibited stronger immunostaining than in the caudal portion (Fig. 4-6). Other TFs were expressed in fractions of the cMVST neuron group (in some cases in a stage-dependent manner). Large proportions of cMVST neurons expressed Esrrg (∼90%), Maf (80–95%), Pou3f1 (40–80%), and Evx2 (80–85%) transcripts. Smaller proportions expressed Lbx1 (∼20%) and Foxp2 (10–30%).

A similar picture was found in the chicken, with virtually all cMVST neurons expressing Lhx1+5 and Esrrg, a very large proportion expressing Maf or Onecut1 (∼90%), and a smaller proportion expressing Foxp2 (∼25%). In contrast to the mouse, essentially no cMVST neurons retrogradely labeled in the chicken at d7.5 or d9 expressed Lbx1 (Fig. 4*C*).

**Figure 7.**
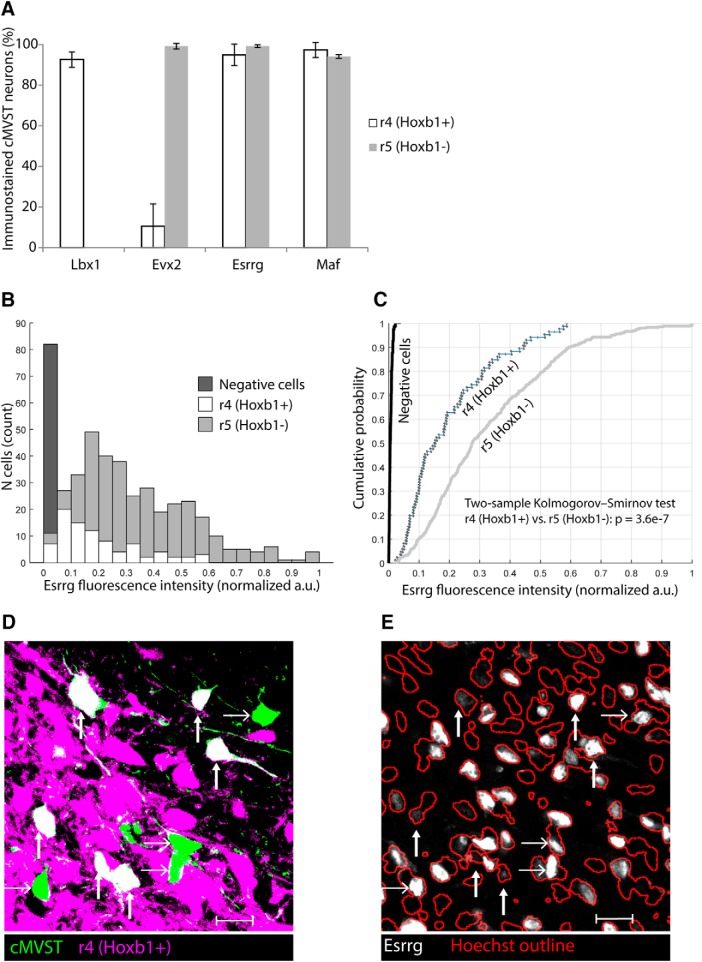
cMVST TFs are differentially expressed in r4- and r5-derived cMVST neurons. ***A***, Percentage of r4-derived (Hoxb1+) versus r5-derived (Hoxb1-) cMVST neurons immunostained for Lbx1, Evx2, Esrrg or Maf (*n* = 2 for Maf, *n* = 3 for others). Lbx1 and Evx2 immunostaining discriminates r4-cMVST and r5-cMVST neurons. ***B***, Histogram of Esrrg fluorescence intensity [in normalized arbitrary units (a.u.)] in individual r4-cMVST neurons (white bars), r5-cMVST neurons (light gray bars), and Esrrg-negative cells (dark gray bar, comprising nearby non-immunostained nuclei), assessed in *n* = 7, 8, and 9 sections from each of *n* = 3 different hindbrains. Note that r4-cMVST neurons are substantially fewer than r5-cMVST neurons, as expected from the relative sizes of the two portions of the cMVST group. ***C***, Empirical cumulative probability distributions of Esrrg fluorescence intensity in the same cMVST neurons and Esrrg-negative cells as in ***B***. Note that using cumulative probability distributions in effect normalizes the numbers of r4- and r5-cMVST neurons. The statistical difference between the two distributions was tested using the two-sample Kolmogorov–Smirnov test, with *p* value as indicated. ***D***, Confocal image of cMVST neurons (green), r4-derived cells (magenta), and dual positive r4-cMVST neurons (white). ***E***, Same field-of-view as in ***D***, with Esrrg-immunostained nuclei (white) and Hoechst stained nuclei (red outlines). ***D***, ***E***, vertical arrows indicate r4-cMVST neurons (mostly weak Esrrg staining), horizontal arrows r5-cMVST neurons (mostly strong Esrrg staining). All quantification was done on sections from E16.5 *b1r4-Cre x tdTomato* mice. Ventral, left; lateral, down. Optical slice thickness, 2 μm. Scale bar, 20 μm.

The above observations suggested that some TFs are common to all cMVST neurons, whereas others might be differentially expressed in the minor r4- and major r5-derived portions of the cMVST. We tested this directly by combining immunohistofluorescence and retrograde labeling in *b1r4-Cre x tdTomato* mice, which we did at E16.5 to ensure substantial retrograde labeling of the cMVST. Lbx1 and Evx2 had regionally restricted immunostaining patterns within the cMVST with a strong relationship to rhombomeric origin: Lbx1+ cMVST neurons were exclusively derived from r4, and Evx2+ cMVST neurons were primarily derived from r5 (Fig. 7*A*, and Fig. 4-3). Only ∼10% of r4-cMVST neurons expressed Evx2, whereas virtually all r5-cMVST neurons expressed Evx2 but not Lbx1 (Fig. 7*A*).

These results reveal TF combinations that distinguish the r4- and r5-derived portions of the cMVST, with the presence or absence of Lbx1 expression providing a clear distinction of the two portions. Thus, the TF combination Lhx1+5/Onecut1,2,3/Lbx1 is restricted to the r4-derived portion of the cMVST, whereas the TF combination Lhx1+5/Onecut1,2,3/Evx2 in the absence of Lbx1 is restricted to the r5-derived part of the cMVST.

Whether these TF combinations are similarly restricted to r4- and r5-cMVST neurons in the chicken was not determined, because of a lack of key antibodies (such as Evx2) and to the relative difficulty in selectively fate-mapping the r4 and r5 lineages in the chicken embryo, which requires additional approaches such as the generation of quail-chicken chimeras (Díaz et al., 1998). However, we note that there may be species-specific differences in cMVST group TF profiles, because we found essentially no Lbx1+ cMVST neurons in the chicken, despite this being a defining feature of the r4-derived portion of the cMVST group in the mouse. On the other hand, this could be because of immaturity of the r4-cMVST subgroup in the chicken at the stages studied (see Discussion).

### TFs that define additional cMVST neuron subpopulations

cMVST neurons that were immunopositive for each of Maf, Esrrg, Foxp2, and Pou3f1 were also differentially distributed, albeit less strikingly than for Evx2 and Lbx1, and they also exhibited some dynamic changes (Figs. 4-3, 4-4).

#### Maf in mouse

In the mouse, Maf-negative cMVST neurons (24%) were found mainly rostrally at E13.5, suggesting that they derived from r4. However, by E15.5 there were almost no Maf-negative cMVST neurons at all (5%), and these were found predominantly in the caudal region. By E16.5 ∼95% of all cMVST neurons were Maf+, with no bias toward either rhombomere (Fig. 7*A*). Thus, Maf expression may be restricted to the r5 lineage initially, but expands beyond that lineage during subsequent development. We found no regional pattern of Maf expression in the chicken cMVST.

#### Esrrg in mouse

For Esrrg, immunonegative and weakly immunopositive neurons were found mostly in the rostral end of the cMVST group, intermingled with strongly immunopositive neurons. Costaining for Esrrg and Evx2 (Evx2 being used as a proxy for r5 origin; see above) at E13.5 and E15.5 showed that approximately half of the r4-cMVST neurons were also Esrrg-negative (E13.5: 39 ± 24% SD, E15.5: 53 ± 5% SD). By E16.5, however, ∼90% of the r4-cMVST neurons were Esrrg+, although the immunostaining was weak in many of them (Fig. 7). Fluorescence intensity measurements confirmed that Esrrg immunostaining was weaker in r4-cMVST neurons compared with r5-cMVST neurons (Fig. 7*B*–*E*). We found no regional pattern of Esrrg expression in the chicken cMVST.

#### Foxp2

In the mouse, Foxp2+ neurons were rare in the rostral portion of the cMVST group at E13.5 and E15.5, whereas the caudal portion contained intermingled Foxp2+ and Foxp2-negative cMVST neurons. The number of Foxp2+ cMVST neurons in the caudal portion of the cMVST group declined noticeably from E13.5 to E15.5 (Fig. 4-3). In the chicken, we found a slight dorsolateral bias in the distribution of Foxp2+ cMVST neurons (Fig. 4-4).

#### Pou3f1 in mouse

In the mouse, Pou3f1 was expressed in both the rostral and caudal portions of the cMVST group in approximately equal proportions at E13.5, but at E15.5 there was a bias toward large numbers of Pou3f1+ neurons toward the mediocaudal pole of the cMVST group (Fig. 4-3). There was a precipitous drop in the proportion of Pou3f1+ cMVST neurons from E13.5 to E15.5 (Fig. 4*B*).

### A restricted, conserved TF signature that defines the r5-cMVST group

#### Spatial restriction of a 4-TF signature

Having identified TF combinations that distinguish the r4- and r5-derived portions of the cMVST group in the mouse, we then asked whether any TF combination represents a conserved TF signature that is restricted to either of these portions of the cMVST group. Here we focus on the r5-cMVST neurons.

**Figure 8.**
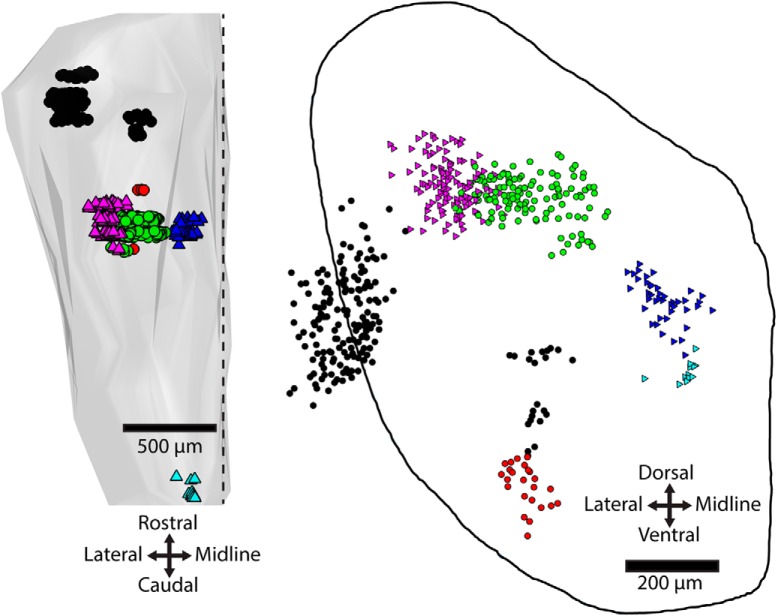
Expression of the Lhx1+5/Evx2/Maf/Esrrg TF combination in the E13.5 mouse is restricted to two clusters of neurons. Only two neuron groups in the mouse CNS coexpressed Lhx1+5/Evx2/Maf/Esrrg, indicated by green circles (cMVST group) and blue triangles (medially extending neighboring neuron population). Retrogradely labeled LVST neurons (magenta triangles) are plotted from alternating serial sections from the same preparation for orientation. The other neuron groups shown (black circles, red circles, turquoise triangles) are Evx2/Maf/Esrrg triple-positive but Lhx1+5-negative. Outline on the right is drawn from the level of the cMVST group. Scale bars: left, 500 μm; right, 200 μm.

Preliminary experiments assessing various 4-TF combinations in r5-cMVST neurons led us to the combination Lhx1+5/Evx2/Maf/Esrrg. Immunofluorescence assessment from lumbar spinal cord to cortex in the E13.5 mouse showed that the only cells that expressed this TF combination were in the location of the cMVST group and extending medially from this group toward the midline (Fig. 8; Table 5).

**Table 5.** Number of Lhx1+5/Evx2/Maf/Esrrg quadruple-positive neurons at different developmental stages in the mouse

** Stage**	**r5-cMVST lateral**	**Medial extension**	***N* preparations**	**Sections assessed/preparation**
E9.5	0	0	3	Every 4th
E11.5	8	2	1	Every 4th
E13.5a	85 ± 25	25 ± 21	3	Every 6th
E15.5b	98 ± 21	25 ± 9	3	Every 6th

Counts of quadruple positive cells ± SD in different areas of the mouse CNS.

*^a^*Counts represent entire CNS. Only the hindbrain was assessed at other stages.

**Figure 9.**
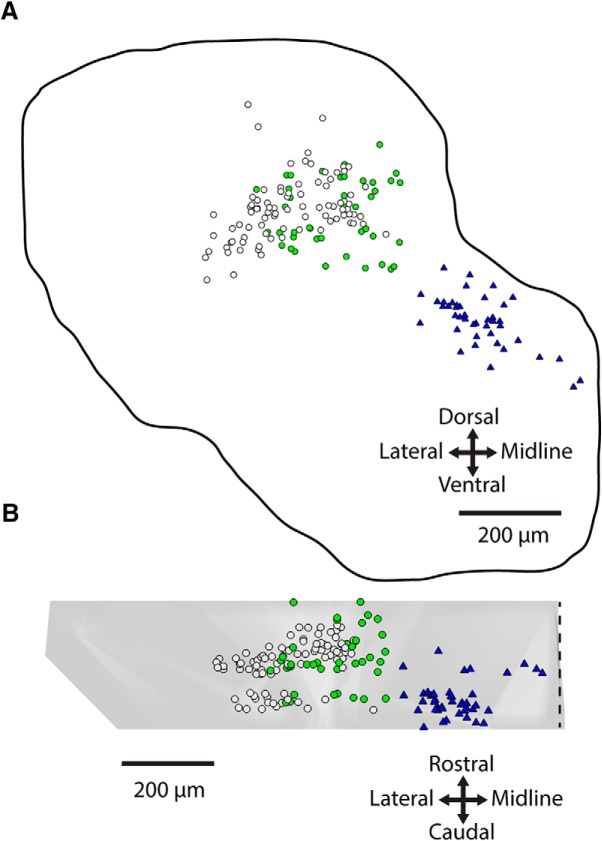
Overlap of retrograde labeling and Evx2/Maf/Esrrg-immunostaining in the region of the cMVST group. ***A***, ***B***, Locations of laterally located Evx2/Maf/Esrrg-expressing cells in an E15.5 mouse that overlap with contralateral retrograde labeling (white circles; *n* = 87) or not (green circles; *n* = 41). Medially located Evx2/Maf/Esrrg-expressing neurons with no retrograde labeling shown as blue triangles (*n* = 42). Neurons from every third section throughout the rostrocaudal extent of the cMVST group shown. ***A***, Transverse section through the level of the cMVST group outlined. ***B***, Side view of the rostrocaudal extent of the cMVST group.

*^b^*Preparations at E15.5 were not coimmunostained for Lhx1+5 because they included retrograde labeling of the cMVST; here, counts represent Evx2/Maf/Esrrg immunostained neurons in and around the cMVST domain only (other brainstem areas were not assessed; Fig. 9).

We could not make the same assessment of the r5-cMVST TF signature in the chicken because of the lack of a functioning Evx2 antibody. Nevertheless, we had already determined that virtually all cMVST neurons in the chicken express Lhx1+5, Maf, and Esrrg, as well as Onecut1. Together with the fact that Evx2 transcripts were detected in chicken cMVST RNA samples (Fig. 3*B*; Table 3), and that no Lbx1 immunopositive neurons were detected in the chicken cMVST, it appears that the Lhx1+5/Evx2/Maf/Esrrg TF signature of r5-cMVST neurons is conserved in the two species.

#### The 4-TF signature cannot be reduced to a 3-TF signature, but one 3-TF signature can be used as a proxy

To enable a direct demonstration through retrograde labeling of the expression of this TF signature by the r5-cMVST neurons, we then assessed the possible 3-TF combinations among Lhx1+5, Evx2, Maf, and Esrrg to determine whether any of these could be used as a proxy for the 4-TF signature. We found that each 3-TF combination was expressed by additional neuron groups within the hindbrain. However, the additional neuron groups expressing the Evx2/Maf/Esrrg combination were well separated from the cMVST group and the contiguous medially extending neuron population (Fig. 8), allowing us to use this 3-TF combination as a proxy. We combined Evx2/Maf/Esrrg immunostaining with retrograde labeling of the cMVST at E15.5 to definitively assess overlap with the r5-cMVST neurons. This demonstrated that the contiguous medially extending neuron population was not retrogradely labeled and thus did not project to the spinal cord, whereas ∼50% (38, 53, and 66% in 3 separate preparations) of neurons in the cMVST domain was retrogradely labeled (Fig. 9). E15.5 is during the period when cMVST axons are growing toward the spinal cord, so some cMVST neurons are likely not retrogradely labeled because their axons have not yet reached C1. Most non-retrogradely labeled Evx2/Maf/Esrrg immunopositive neurons were positioned within the cMVST group domain, intermingled with retrogradely labeled cMVST neurons (Fig. 9). The proportion of r5-cMVST neurons expressing the Evx2/Maf/Esrrg signature was 89% (the remainder lacking either Maf or Esrrg; compare Fig. 4).

We have not yet determined the identity of the neurons that extend medially from the cMVST group, nor have we identified alternative TF signatures that differentiate them from the cMVST neurons.

#### Developmental appearance of the 4-TF signature

Having established that Lhx1+5/Evx2/Maf/Esrrg expression was restricted to the r5-cMVST neuron group and its contiguous, medially extending neighbor group, we set out to assess how early this 4-TF signature appears during development. Quadruple-positive r5-cMVST neurons were not present at E9.5 (because there is yet no Maf or Esrrg expression; see description of LVST-related TF development), but 10 such neurons were seen in a preparation at E11.5 (Table 5), 8 of which were located laterally (in the presumptive cMVST group domain) and 2 of which were found medially, close to the midline. By E13.5 the number of quadruple-positive neurons had increased in both lateral and medial regions to levels seen in retrogradely labeled preparations at E15.5 (Table 5).

### In search of a TF signature for the r4-cMVST group

**Figure 10.**
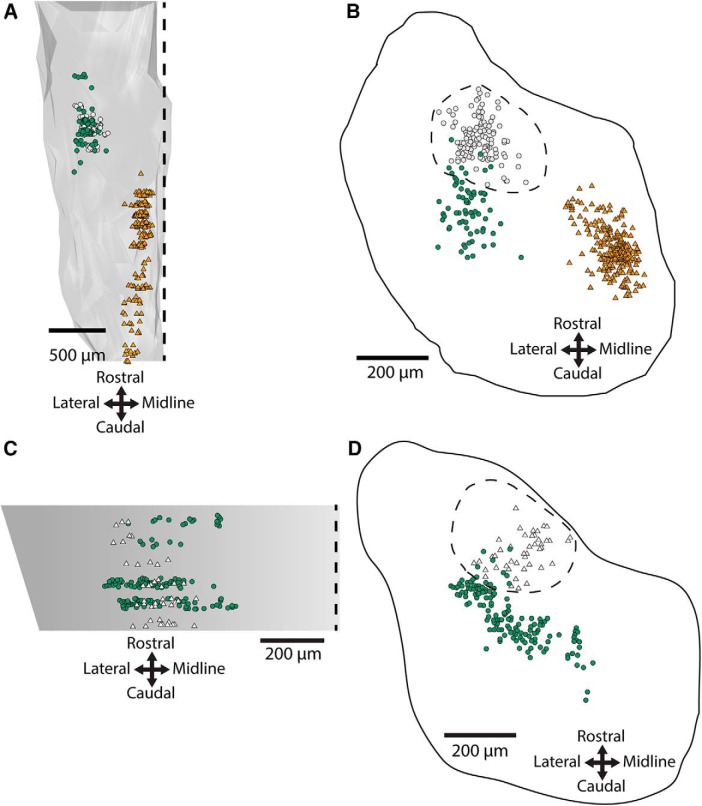
Hindbrain cells expressing Lbx1/Lhx1+5/Onecut2/Maf are not restricted to the r4-cMVST group. ***A***, 3D map and (***B***) transverse projection map of cells coexpressing Lbx1/Lhx1+5/Maf/Onecut2 in the E13.5 mouse hindbrain, with retrogradely labeled LVST neurons (white circles) plotted from adjacent interleaved sections for orientation. Green circles and orange triangles represent Lbx1/Lhx1+5/Maf/Onecut2+ cells located, respectively, at the same rostrocaudal level but predominantly ventral to the cMVST and LVST, and at a more caudal and ventromedial location. The dashed line roughly delineates the location of LVST and cMVST neurons, and contains only a few quadruple-immunolabeled cells. Cells are plotted from every sixth serial transverse section; in ***B*** these are projected onto a single plane. ***C***, 3D map and (***D***) transverse projection map of cells coexpressing Lbx1/Lhx1+5/Onecut2/tdTomato in the E14.5 *b1r4-Cre x tdTomato* mouse hindbrain, with retrogradely labeled cMVST neurons (white triangles) plotted from adjacent interleaved sections for orientation. Green circles represent Lbx1/Lhx1+5/Onecut2/tdTomato+ cells. The 3D reconstruction in ***A*** is limited rostrocaudally to the level of the quadruple-immunolabeled cells and the cMVST neurons. The dashed line roughly delineates the location of LVST and cMVST neurons, and contains 11 quadruple-immunolabeled cells that are likely to be r4-cMVST neurons. Typically 20% of the cMVST group as a whole (45 neurons in this reconstruction) are r4-cMVST neurons. This corresponds well with the counts of quadruple-labeled cells here (45 × 0.2 = 9). Cells are plotted from every fourth serial transverse section; in ***D*** these are projected onto a single plane.

Identifying a TF signature restricted to the r4-cMVST group posed more of a challenge because only three TFs were robust markers of this subgroup: Lbx1, Lhx1+5, and Onecut1/2/3. As mentioned earlier in the section entitled *Esrrg in mouse*, Esrrg was only detected in ∼50% of r4-cMVST neurons, and at least some r4-cMVST neurons are Maf-negative. We therefore assessed the 4-TF signature Lbx1/Lhx1+5/Maf/Onecut, using Onecut2 as a proxy for Onecut1 and 3, because Onecut family members are usually coexpressed, and our Onecut1 and 3 antibodies were of the same host species as our Lbx1 antibody. Coimmunostaining in the E13.5 mouse hindbrain revealed that the majority of neurons expressing this 4-TF signature were not r4-cMVST neurons, or even cMVST neurons at all (Fig. 10*A*,*B*). Two distinct populations of neurons were observed, with different rostrocaudal and dorsoventral locations. The largest and more caudal of these was clearly non-vestibular, as it was located ventromedially (Fig. 10*A*,*B*, orange triangles). The more rostral population contained some r4-cMVST neurons, but the majority was non-cMVST neurons, as they lay well outside the cMVST group domain (Fig. 10*A*,*B*, green circles). We then asked whether these rostral non-cMVST neurons were derived from r4, by immunostaining for Lbx1/Lhx1+5/Onecut2 in the *b1r4-Cre x tdTomato* mouse. If not, then this 4-TF signature might be unique to the r4-cMVST group within the r4 lineage. Nearly all the Lbx1/Lhx1+5/Onecut2-positive cells in this area were, however, colabeled for tdTomato, indicating that they derived from r4 (Fig. 10*C*,*D*). We conclude that this 4-TF signature, although it labels some r4-cMVST neurons, is not unique to the r4-cMVST, even within r4. We therefore did not assess it in the rest of the CNS.

## Discussion

### Principal findings

We have previously described that the vestibulospinal system comprises three coherent groups of neurons with distinct anatomic locations, developmental origins, projection patterns and functional connectivity (Glover and Petursdottir, 1988, 1991; Díaz and Glover, 2002; Díaz et al., 1998; Auclair et al., 1999; Glover, 2000a; Pasqualetti et al., 2007; Kasumacic et al., 2010; Di Bonito et al., 2015; Lambert et al., 2016). Specifically, the two largest of these groups, the LVST and cMVST groups, have respectively dorsolateral versus dorsomedial locations, r4 versus r4+r5 origins, ipsilateral versus contralateral projections, and synaptic targets along the entire length of the spinal cord versus limited to the cervical spinal cord. Although previous studies have documented the expression of Phox2b and Lbx1 in LVST neurons (Schubert et al., 2001; Chen et al., 2012), a more complete assessment of TF profiles in VS neurons has been lacking. Here we use RNAseq to provide the first comprehensive post-mitotic transcriptomes of the LVST and cMVST neuron groups, in two species representing two different vertebrate classes. We further demonstrate protein expression of a number of TFs by immunohistofluorescence, and identify conserved sets of TFs that distinguish the LVST group from the cMVST group, and the r4-derived portion of the cMVST group from the r5-derived portion. Using combinatorial TF immunostaining and inspection along the entire length of the CNS, we identify two specific TF signatures that are restricted to respectively the LVST group and the r5-cMVST group, together with a smaller group of nearby neurons in each case. Finally, we identify additional TFs that define subpopulations within the LVST and cMVST neuron groups. Together, these results demonstrate a molecular underpinning that correlates with and likely contributes to the anatomic and functional characteristics that distinguish these brainstem-to-spinal cord projection neuron groups.

### Methodological considerations

Neurons acquire cell-specific phenotypes through transcriptional multi-step cascades during development, and transcriptional control relies heavily on differential TF expression (Jessell, 2000; Hobert, 2008; Ooi and Wood, 2008; Achim et al., 2014; Kessaris et al., 2014; Arendt et al., 2016). To ascertain which TFs are expressed in nascent post-mitotic VS neurons, we used RNAseq on manually sorted, retrogradely labeled neurons, which ensured selective isolation of the relatively few neurons involved (Hempel et al., 2007; Okaty et al., 2011). By investigating the VS transcriptome in both mouse and chicken, we could hone in on phylogenetically conserved TF signatures.

TFs that specify the identity of neuronal lineages are often rapidly downregulated in postmitotic neurons, which typically begin to express new sets of cell-type-specific TFs (Achim et al., 2014). Here, we had to retrogradely label postmitotic VS neurons to identify and isolate them. This restricted the time window in which we could assess transcriptomic profiles. Chen et al. (2012), using a Hoxb1-GFP mouse, observed putative LVST axons extending toward the spinal cord by E10.5. Here we found that the LVST neuron group can be retrogradely labeled from the spinal cord by E11.5 and d4–d5 (HH24-26), and the cMVST neuron group can be retrogradely labeled from the mid-medulla by E13.5 and d7.5–d8.5 (HH30-32), in the mouse and chicken embryo respectively. We could obtain sufficient numbers of cMVST neurons only by retrogradely labeling from the mid-medulla at E13.5/d7.5 (see Materials and Methods). To minimize sample variation within species because of developmental differences, we thus chose E13.5 and d7.5 for harvesting both the LVST neurons (labeled from C1) and the cMVST neurons (labeled from mid-medulla). Based on the above indications of the earliest time of axon outgrowth, and presuming that axon outgrowth begins shortly after the neurons are born, our RNAseq data represent expression at least 3 d after the LVST neurons are born. Because the cMVST neurons appear to develop ∼2 d later than the LVST, we estimate that the RNAseq data represent expression at least 1 d after the cMVST neurons are born. All of the TFs that make up the VS group-specific signatures were expressed at both E13.5/d7.5 and E15.5/d9, and as early as E11.5 in the mouse, indicating that the signatures are valid over a broad temporal range. The extent to which these TFs or others that we have captured by RNAseq are involved in lineage specification versus cell differentiation (or both) remains an open question.

### Commonality and exclusivity in vestibulospinal TF signatures

#### Phox2b versus Lhx1+5 as discriminants of the LVST and cMVST groups

Phox2b and Lhx1+5 are mutually exclusive, discriminating the LVST from the cMVST. Interestingly, forced expression of *Phox2* genes downregulates Lhx1+5 in the spinal cord *in vivo* and also suppresses the growth of commissural axons (Hirsch et al., 2007; Pla et al., 2008), suggesting that repressive interactions between these two TFs may be involved in specifying the LVST from the cMVST.

Phox2b has been described as a pan-visceral homeodomain TF, because most Phox2b-expressing neurons are associated with visceral function, e.g., branchial and visceral motoneurons (Pattyn et al., 2000), and first- and second-order visceral sensory neurons (D’Autréaux et al., 2011). In the adult rat, however, some neurons with no relationship to autonomic function express Phox2b (Kang et al., 2007). Our finding that all LVST neurons express Phox2b at embryonic stages is further evidence of *Phox2b* expression outside of traditionally defined visceral neurons, raising interesting questions about the developmental role of Phox2b, the definition of visceral function, and the evolution of the LVST neuron phenotype.

Lhx1 and Lhx5 are expressed in a number of different cell types throughout the CNS. The antibody we and many other studies have used does not discriminate between the two, but evidence suggests that they are often coexpressed (Cepeda-Nieto et al., 2005; Moreno et al., 2005; Pillai et al., 2007). Lhx1+5 and other Lim homeodomain TFs have been shown to be essential for proper differentiation, axon guidance, and neurotransmitter phenotype of diverse neuron types, and Lhx1+5 is expressed by some reticulospinal neurons (Kania et al., 2000; Cepeda-Nieto et al., 2005; Pillai et al., 2007; Zhao et al., 2007; Bröhl et al., 2008; Kohl et al., 2015), although a relationship between these and the cMVST group is not immediately obvious.

#### Lbx1 and Evx2 discriminate r4- and r5-derived VS neurons

Lbx1 was expressed in all LVST neurons in both mouse and chicken and 90% of neurons in the r4-cMVST group in the mouse. In the chicken, in which r4- versus r5-derived neurons could not be distinguished because we did not include rhombomeric fate-mapping, we found no Lbx1+ cMVST neurons. Because r4-derived neurons are known to exist in the chick (Díaz et al., 1998), we believe that this discrepancy relates to the timing of their axon outgrowth to the spinal cord, that is, that we have not retrogradely labeled late enough to detect them. Lbx1 is expressed by a diverse collection of CNS neurons, categorized by some studies as having primarily somatic functions, including somatosensory association interneurons in the dorsal spinal cord (Gross et al., 2002) and somatosensory relay neurons in the trigeminal nucleus (Sieber et al., 2007).

The r5-cMVST group did not express Lbx1, but Evx2. Evx2 is expressed postmitotically in V0 interneurons in the spinal cord and in more extensive neuron populations in the hindbrain and midbrain (Moran-Rivard et al., 2001; Inamata and Shirasaki, 2014). Evx2-expressing neurons are primarily, but not exclusively, commissural (Lu et al., 2015), which is generally consistent with the cMVST phenotype.

#### Maf and Esrrg are VS-common TFs

Among the TFs we studied, Maf and Esrrg are expressed by the vast majority of both LVST and cMVST neurons. These TFs are therefore pivotal for distinguishing VS neurons from other neurons that express combinations of Phox2b, Lbx1, Evx2 and Lhx1+5. Exceptions were the somewhat lower proportions of Esrrg+ neurons in the dorsolateral part of the LVST group in chicken and in the r4-cMVST group in mouse. Both Maf and Esrrg play critical roles in specifying post-mitotic cell types, both within and outside the nervous system (Maf: Wende et al., 2012; Zhang and Guo, 2015; Esrrg: Süsens et al., 2000; Friese et al., 2009; Patthey et al., 2016; Yoshihara et al., 2016). Maf belongs to the activated protein-1 superfamily (Zhang and Guo, 2015). Its paralog MafB is selectively expressed in r5 and r6 in mice and zebrafish, highlighting the relationship of Maf genes to hindbrain development (Moens and Prince, 2002; Giudicelli et al., 2003). Esrrg is a constitutively active nuclear hormone receptor, with no known physiologic activating ligand (Huppunen et al., 2004). Expression of Esrrg and its invertebrate homolog is also rhombomere-specific in developing zebrafish and amphioxus (Bardet et al., 2005).

### CNS-wide restriction of identified VS TF signatures

Increasing efforts are being made to identify TF signatures for functionally identifiable neuron groups within the motor system. Recent evidence suggests a much greater diversity in the functional organization of brainstem-to-spinal cord projections than was previously recognized (for review, see Perreault and Glover, 2013; Brownstone and Chopek, 2018), but to date there have been few attempts to codify this diversity in terms of TF signatures (Cepeda-Nieto et al., 2005; Chen et al., 2012; Bretzner and Brownstone, 2013). One important question that is not often addressed is whether TF signatures are restricted to a neuron group of interest or also expressed elsewhere in the CNS.

Through CNS-wide immunofluorescence mapping in the mouse, we were able to assess the degree to which the TF signatures we identified are specific to the LVST and cMVST neuron groups. The combination of Phox2b/Lbx1/Maf/Esrrg was restricted to the LVST group and a smaller group of neurons that was slightly caudal to the LVST group, had weaker levels of Maf expression, and did not originate from r4. The combination of Evx2/Lhx1+5/Maf/Esrrg was restricted to the r5-cMVST group and to more medial neurons at the same rostrocaudal level. In neither case were the vicinal non-LVST or non-r5-cMVST neurons retrogradely labeled from the spinal cord at the developmental stages examined. Whether this means they never project to the spinal cord, or are in fact nascent bulbospinal neurons whose axons have not yet reached the spinal cord, remains to be determined. Despite the fact that they also are expressed by additional neurons near the LVST and cMVST groups, it is notable that these TF signatures are otherwise unique within the CNS at the stages examined.

In contrast to the LVST and r5-cMVST neuron groups, we have not yet found a TF signature that is similarly restricted to the r4-cMVST group. The 3-TF signature that we found that provides full coverage of the r4-cMVST group, Lbx1/Lhx1+5/Onecut2, was expressed by many other cell groups within the CNS. Even when assessed only within the r4-domain, many non-cMVST neurons within r4 expressed this signature.

Further work is required to determine whether additional TFs distinguish the LVST and cMVST groups from the smaller neighboring non-VST neurons. Nevertheless, the degree of restriction exhibited makes these 4-TF signatures useful markers for the groups and should facilitate further investigations into vestibulospinal development and function.

### Proposed progenitor origins and evolutionary implications

The differential expression of TF signatures by the LVST and cMVST groups are related to rhombomeric origin, and thus to rhombomere-specific TFs involved in anteroposterior-patterning. Indeed, Hoxb1 is a critical factor in specifying the LVST and r4-cMVST neuron phenotypes; in the absence of Hoxb1 expression these groups do not develop (Di Bonito et al., 2015). Which r5-related Hox TF(s) is(are) similarly critical for the specification of the r5-cMVST group remains to be determined, but is likely to be among Hoxa3/b3/d3 (Keynes and Krumlauf, 1994).

Although largely composed of postmitotic TFs, the signatures indicate a composite origin of the vestibulospinal system with respect to dorsoventral neural progenitor domains. Expression of Phox2b and Lbx1 in the hindbrain is specific for the dB2 progenitor domain (Hernandez-Miranda et al., 2017) indicating that this is the origin of the LVST group, as previously proposed by Chen et al. (2012). The dB2 progenitor domain is unique to the hindbrain, suggesting that there is no LVST neuron group homolog elsewhere.

By contrast, the expression of Lbx1 and Lhx1+5 in the r4-cMVST neurons suggests a possible origin from either the pdB1, the pdB4 or the late pdBLa progenitor domains (Hernandez-Miranda et al., 2017). Currently unpublished work from our laboratories indicates that none of the cMVST neurons derive from Ascl1-expressing progenitors (Glover and Dymecki, unpublished observations), suggesting an origin from the pdB4 domain.

The r5-cMVST group TF signature suggests yet another DV origin, given the inclusion of Evx2 and the exclusion of Lbx1. Evx2 is indicative of a p0 progenitor origin, which would place the r5-cMVST group in the unusual position of deriving from a ventral as opposed to a dorsal progenitor domain.

That these diverse lineages converge on a vestibulospinal phenotype raises interesting questions about the evolution of the vestibulospinal system. Given that they derive from progenitor domains that are found throughout the hindbrain-spinal cord axis, it would seem that the r4- and r5-cMVST groups represent a situation in which spinal interneuron populations have been co-opted and repurposed for vestibulospinal function. Because the cMVST is primarily involved in vestibulo-collic reflexes (that is, reflexive movements of the head about the neck), this co-option would most likely have occurred first with the advent of a movable neck, that is, later than fish and amphibians. By contrast, the LVST group is among vestibulospinal neurons uniquely related to Phox2b, and projects to the entire length of the spinal cord in both limbed and non-limbed vertebrates. Given that Phox2 genes predate the chordates, and that balance-related control of both trunk and limb musculature is important throughout the vertebrate radiation, this could mean that the LVST is the primordial vestibulospinal projection, which has been supplemented by the cMVST (and the iMVST) later in evolution.

### VS group subpopulations defined by additional TFs

The cMVST group is composed of two subgroups based on rhombomeric origin. Both the LVST group and the cMVST group are likely to exhibit further heterogeneity, given that they target various spinal segments and neuron subtypes (Wilson and Yoshida, 1969; Uchino and Kushiro, 2011; Kasumacic et al., 2015), and receive various types of vestibular afferent input (Boyle and Pompeiano, 1981; Uchino and Kushiro, 2011). Expression of TFs that define subpopulations within these groups was therefore expected.

We found that Lbx1, Evx2, Onecut1/2/3, Foxp2, Pou3f1, and to a certain degree Maf and Esrrg, all defined subpopulations of neurons within at least one VS group. How these expression patterns relate to functional differences among VS neurons remains to be determined. Some predictions can be made, however. Several studies have shown in mammals that respectively rostral versus caudal LVST neurons target cervical versus lumbar spinal levels (Brodal, 1963; Shamboul, 1980; Esposito et al., 2014). This could be related to our observation that Pouf31 is expressed in more rostral LVST neurons, whereas Onecut1/2/3 are expressed in more caudal LVST neurons. Similarly, Foxp2 (which was absent in the LVST group) was expressed by more neurons in the caudal and dorsolateral portions, respectively, of the mouse and chicken cMVST neuron group.

We note that such intrinsic patterning within VS neuron groups could be explained by transitory developmental states, rather than permanent functional subdivisions, although some examples (such as the rostrocaudally biased Onecut and Foxp2 expression patterns) are stable during the developmental period we have studied. Clearly, further work is needed to determine the relevance of these TFs for functional diversity within the VS groups.

### Significance of the work

The molecular mechanisms that specify hodological and anatomic subdivisions within brainstem-to-spinal cord projection neurons are poorly understood. Assessment of TF expression profiles provides information essential for addressing this question. Here we show that TF signatures conserved between mammals and birds can be defined for specific, functionally identifiable VS projection neuron groups.

This lends support to the idea that descending systems for communication between the brainstem and spinal cord are set up by a genetic blueprint established early in vertebrate evolution, in this case at least 300 million years ago, when the avian and mammalian lineages diverged.

The identification of specific TF signatures provides opportunities in several directions, including to ascertain evolutionary relationships and changes, to unravel functional heterogeneity, to facilitate molecular manipulations, to elucidate molecular programs of differentiation and identify terminal selector genes, and to generate specific types of vestibulospinal neurons from stem or progenitor cells *in vitro* for research and medical purposes.

## Synthesis

Reviewing Editor: Muriel Thoby-Brisson, CNRS UMR 5287 Université Bordeaux Decisions are customarily a result of the Reviewing Editor and the peer reviewers coming together and discussing their recommendations until a consensus is reached. When revisions are invited, a fact-based synthesis statement explaining their decision and outlining what is needed to prepare a revision will be listed below. The following reviewer(s) agreed to reveal their identity: Carmen Birchmeier.

General comments:

This manuscript takes a challenging, yet methodical approach to identifying unique combinations of transcription factors that uniquely label two segments of the vestibulospinal tract within the brain at specific developmental points, the LVST and cMVST. The authors first use unbiased transcriptomics to identify candidate transcription factors enriched within each of these tracts and then use combinatorial immunofluorescence to define a panel of four transcription factors that uniquely labels each within the brain. Thus neurons that project along the lateral and medial  vestibulospinal tract  (LVST and cMVST, respectively) are characterized in molecular terms by RNA sequencing after retrograde labeling in fetal mice and chick. These neurons receive direct input from the vestibular system, and innervate directly and indirectly motor neurons in the spinal cord. Thus, they play an important role in the control of posture and movement. In addition, the receive input from the cerebellum and from other brainstem regions and thus integrate additional information important for motor control. The manuscript has a focused hypothesis (that each tract can be uniquely labeled with a combination of transcription factors) and thoroughly characterizes the expression patterns of these markers throughout the hindbrain and spinal cord.

This study certainly warrants publication and will provide the foundation for future studies to 1) define the role of these genes in development of the VST and 2) determine if the quartets of markers are uniquely expressed throughout all development and can therefore be used as true lineage markers for small populations of specific neural types. This study defines a workflow that others could mimic in order to identify unique and specific markers that will enable them to genetically manipulate neurons that have unique properties and projection patterns. The quality of the data is very high. Very few molecular data for these neuronal types exist, and the results of the manuscript are thus very useful for researchers interested in movement control. The availability of clear markers for these important neuron types will in the future allow a better analysis of this neuronal type; it also facilitates manipulation using targeted genetics for further functional analyses.

Minor comments :

1. Is the retrograde tracing of VS neurons by spinal cord projections specific within the brainstem regions investigated? This could be easily demonstrated with a classic neuroanatomy stain such as a Nissl stain on the same tissue section that is fluorescently retrogradely labeled from the spinal cord.

2. Please show the data for the co-staining with Phox2b/Lbx1/Maf/Esrrg to uniquely identify the LVST within the brain. This data is only described and never shown. This could be incorporated into Figure 5.

3. Although it is clear from the quantification that Lbx1 and Evx2 can distinguish r4 and r5 cMVST groups, the data for ESRRG intensity is not nearly as compelling. It is clear that ESRRG doesn't absolutely define these rhombomeres and that perhaps if more r4 cells were quantified, then the intensity distributions would overlap even more. If included, quantify a similar number of r4 and r5 neurons.

4. Figure 10C. is impossible to assess since the quality of the image is poor. Furthermore, it is unclear why the markers Lhx1+5/Onecut2/Lbx1 do not overlap within the dashed circle demarking the cMVST. My understanding from the text (page 26) is that these markers were chosen because they label all of the neurons in the r4-cMVST. Additionally, the r4-cre line does not appear lineage trace this domain (10D). Please clarify panels C-E and provide clear data.

5. Lines 580/581 are unclear to me and should be rephrased: Our RNAseq data thus represent expression at least 3 days and 1 day post-mitotic for  the LVST and cMVST neurons, respectively.  

6. It is unclear to me how Fig. 1B was generated- please explain in more depth.

## Author Response

Dear Muriel,

We enclose our revised manuscript, and provide below our responses to the reviewers´ comments and describe the changes we have made accordingly. When it comes to changes to figures, associated modifications have been made in the main text and figure legends.

We have also now included citations to multiple references that were removed for anonymization in connection with the review process.

We have added a statement on availability of RNAseq data files in the RNAseq methods section, currently with a placeholder accession number. We anticipate receiving the accession from GEO in the next few days, and hope this can be added in the proofing stage. (text: “RNAseq data files have been uploaded to the Gene Expression Omnibus database (https://www.ncbi.nlm.nih.gov/geo/).

We hope that the manuscript is now “up to snuff” and acceptable for publication.

Thanks very much for your efforts and the efforts of the reviewers.

Cheers,

Minor comments:

1. Is the retrograde tracing of VS neurons by spinal cord projections specific within the brainstem regions investigated? This could be easily demonstrated with a classic neuroanatomy stain such as a Nissl stain on the same tissue section that is fluorescently retrogradely labeled from the spinal cord.

We are a little uncertain about what the question is here. If the question is if the LVST and cMVST are specific groups within the vestibular region, or even the hindbrain (or entire brain, for that matter), then the answer is yes. This is based on our investigations of these VS groups over the past 30 years (we identified and described them first in 1988). The basis for this is not immediately evident in the anonymized manuscript, but after de-anonymization several relevant references will be cited from our previous work that document this point. The LVST and cMVST groups are unique, coherent neuron groups within the VS system and have no obvious homologs in other projection systems. They have specific developmental origins, cytoarchitectonic relationships, projection pathways, and patterns of synaptic connections in the spinal cord. They can be selectively labeled with retrograde tracing once their projection pathways are known (they were first identified and characterized through a combination of retrograde tracing and tract cutting). They are also highly conserved among vertebrates (we have labeled and characterized them in mammals, birds, frogs and fish - and more recently snakes).

As it turns out, it is not as simple as suggested to combine retrograde labeling and Nissl staining to make this point. We devoted an entire study to a direct comparison of retrogradely labeling of VS neurons (and other vestibular projection neurons) and cytoarchitectonics in the same brainstems (of the chicken embryo - Diaz et al (2003) J Comp Neurol 457:87). This was a major endeavour that took 2 years to accomplish, and in fact is the only study we know of that has assessed retrograde labeling and cytoarchitectonics of any descending projection system directly in the same brainstem. In this paper, we make the important point that because cytoarchitectonic boundaries are subjective and in many cases prone to varying interpretation, it is not straightforward, without appropriate 3D reconstructions of different sectioning planes, to define how a retrogradely labeled neuron group fits into a cytoarchitectonic system. So we prefer here to allow our previous work to speak to this issue, rather than attempt to replicate that effort in a way that would not be compelling in the present paper.

2. Please show the data for the co-staining with Phox2b/Lbx1/Maf/Esrrg to uniquely identify the LVST within the brain. This data is only described and never shown. This could be incorporated into Figure 5.

Here we have to apologize for not presenting this clearly enough. In fact, the neurons presented in Figure 5A are those that co-express Phox2b/Lbx1/Maf/Esrrg. The point is that within the hindbrain, these are identical to the neurons that express only Phox2b/Maf/Esrrg. It is only when looking outside the hindbrain that one sees that the 3-TF signature Phox2b/Maf/Esrrg is also expressed by other neurons, namely a very medial neuron group in the midbrain. This was shown in Extended Figure 5-1 in the original manuscript. We have now incorporated that figure into Figure 5 (as panel E), which we hope now shows clearly how the expression of the four possible combinations of 3-TFs relate to the parent 4-TF signature Phox2b/Lbx1/Maf/Esrrg.

3. Although it is clear from the quantification that Lbx1 and Evx2 can distinguish r4 and r5 cMVST groups, the data for ESRRG intensity is not nearly as compelling. It is clear that ESRRG doesn't absolutely define these rhombomeres and that perhaps if more r4 cells were quantified, then the intensity distributions would overlap even more. If included, quantify a similar number of r4 and r5 neurons.

The reason that the difference between ESRRG staining intensity in r4- and r5-cMVST neurons does not seem compelling is that the intensity distributions in each population are log normal, and thus overlap to a large extent. Our statistical test of the difference was moreover based on the means, and as required by the condition of independent samples was done on the basis of different hindbrains (n=3, color-coded in the original Figure 7B), not different neurons (n= much more). It is also important to be aware that the number of r4-cMVST neurons in each hindbrain is intrinsically lower than the number of r5-cMVST neurons, because the r4-derived population is only 20% the size of the r5-derived population. It is not kosher to simply increase the number of r4-cMVST neurons to make the populations equal before statistical testing. What we have done instead is to present the raw counts in a different way, namely as histograms, and then as cumulative distributions (new Figure 7C and 7D). The difference between the cumulative distributions can then be tested using the Kolmogorov-Smirnov test, which is actually a more appropriate test in this case. We think that when seen in this way the difference both visually and statistically is convincing.

4. Figure 10C is impossible to assess since the quality of the image is poor. Furthermore, it is unclear why the markers Lhx1+5/Onecut2/Lbx1 do not overlap within the dashed circle demarking the cMVST. My understanding from the text (page 26) is that these markers were chosen because they label all of the neurons in the r4-cMVST. Additionally, the r4-cre line does not appear lineage trace this domain (10D). Please clarify panels C-E and provide clear data.

We apologize for the quality of images in Figure 10C-E. In going through this figure again, we realized that not only were the images difficult to comprehend, but but the presentation was not very intuitive, primarily because only a single section was shown, in which very few Lhx1+5/Onecut2/Lbx1-positive neurons are found within the cMVST domain. We have now restructured Figure 10, and chosen to present a plot of several sections projected onto the transverse plane instead of a photomicrograph of a single section, so that the relationship of these cells to the cMVST is clearer. We remind that the comparison is with the r4-derived part of the cMVST, which is only 20% of the total cMVST. In the figure, the cMVST domain (now enclosed by a dashed circle) contains both r4- and r5-cMVST neurons, so one cannot expect that Lhx1+5/Onecut2/Lbx1 staining will label more than about 20% of these. This also explains why the r4-cre line only labels a similarly minor portion of the domain (with the reporter tdTomato).

So, what we have done in the revised version of Figure 10 is to replace panels 10C-E with two panels (10C and D) that show plots of all Lhx1+5/Onecut2/Lbx1/tdTomato+ neurons and all cMVST neurons in several sections projected onto a single plane, indicated with two different markers. The revised legend explains that about 11 Lhx1+5/Onecut2/Lbx1/tdTomato+ neurons are counted within the cMVST domain (plotted from every fourth section, so the full number would be about 4 times greater), which corresponds well to the expected number based on the count of total cMVST neurons in the projection (45 cMVST cells * 0.2 = 9, again plotted from every fourth section).

To further clarify the figure, in panel 10A,B we use only green circles for all the neurons that were originally depicted separately with blue triangles and green circles. Originally we used blue triangles to depict the Lbx1/ Lhx1+5/Maf/Onecut2-positive neurons inside the cMVST domain and green circles to depict the Lbx1/ Lhx1+5/Maf/Onecut2-positive neurons outside the cMVST domain. Now we use the same symbols, since they represent the same TF signature, and use the dashed line to delineate the cMVST domain.

5. Lines 580/581 are unclear and should be rephrased: Our RNAseq data thus represent expression at least 3 days and 1 day post-mitotic for the LVST and cMVST neurons, respectively.

We have changed the text to the following, and hope that it is now understandable:

To minimize sample variation within species due to developmental differences, we thus chose E13.5 and d7.5 for harvesting both the LVST neurons (labeled from C1) and the cMVST neurons (labeled from mid-medulla) neurons. Based on the above indications of the earliest time of axon outgrowth, and presuming that axon outgrowth begins shortly after the neurons are born, our RNAseq data represents expression at least 3 days after birth for the LVST neurons. Since the cMVST neurons appear to develop about 2 days later than the LVST, we estimate that the RNAseq data represents expression at least 1 day after birth for the cMVST neurons.

6. It is unclear how Fig. 1B was generated- please explain in more depth.

## References

[B1] Achim K, Salminen M, Partanen J (2014) Mechanisms regulating GABAergic neuron development. Cell Mol Life Sci 71:1395–1415. 10.1007/s00018-013-1501-3 24196748 PMC11113277

[B2] Akaike T (1983) Neuronal organization of the vestibulospinal system in the cat. Brain Res 259:217–227. 10.1016/0006-8993(83)91252-0 6297669

[B3] Arendt D, Musser JM, Baker CVH, Bergman A, Cepko C, Erwin DH, Pavlicev M, Schlosser G, Widder S, Laubichler MD, Wagner GP (2016) The origin and evolution of cell types. Nat Rev Genetics 17:744–757. 10.1038/nrg.2016.127 27818507

[B4] Auclair F, Marchand R, Glover JC (1999) Regional patterning of reticulospinal and vestibulospinal neurons in the hindbrain of mouse and rat embryos. J Comp Neur 411:288–300. 10.1002/(SICI)1096-9861(19990823)411:23.0.CO;2-U 10404254

[B5] Bardet PL, Schubert M, Horard B, Holland LZ, Laudet V, Holland ND, Vanacker JM (2005) Expression of estrogen-receptor related receptors in amphioxus and zebrafish: implications for the evolution of posterior brain segmentation at the invertebrate-to-vertebrate transition. Evol Dev 7:223–233. 10.1111/j.1525-142X.2005.05025.x 15876195

[B6] Boyle R, Pompeiano O (1981) Convergence and interaction of neck and macular vestibular inputs on vestibulospinal neurons. J Neurophysiol 45:852–868. 10.1152/jn.1981.45.5.852 7241173

[B7] Bretzner F, Brownstone RM (2013) Lhx3-Chx10 reticulospinal neurons in locomotor circuits. J Neurosci 33:14681–14692. 10.1523/JNEUROSCI.5231-12.2013 24027269 PMC6705172

[B8] Brodal A (1963) Anatomical observations on the vestibular nuclei, with special reference to their relations to the spinal cord and the cerebellum. Acta Otolaryngol Suppl 192:124. 14222372

[B9] Bröhl D, Strehle M, Wende H, Hori K, Bormuth I, Nave KA, Müller T, Birchmeier C (2008) A transcriptional network coordinately determines transmitter and peptidergic fate in the dorsal spinal cord. Dev Biol 322:381–393. 10.1016/j.ydbio.2008.08.002 18721803

[B10] Brownstone RM, Chopek JW (2018) Reticulospinal systems for tuning motor commands. Front Neural Circuits 12:30. 10.3389/fncir.2018.00030 29720934 PMC5915564

[B11] Cepeda-Nieto AC, Pfaff SL, Varela EA (2005) Homeodomain transcription factors in the development of subsets of hindbrain reticulospinal neurons. Moll Cell Neurosci 28:30–41. 10.1016/j.mcn.2004.06.016 15607939

[B12] Chen Y, Takano-Maruyama M, Fritzsch B, Gaufo GO (2012) Hoxb1 controls anteroposterior identity of vestibular projection neurons. PloS One 7:e34762. 10.1371/journal.pone.0034762 22485187 PMC3317634

[B13] D’Autréaux F, Coppola E, Hirsch MR, Birchmeier C, Brunet JF (2011) Homeoprotein Phox2b commands a somatic-to-visceral switch in cranial sensory pathways. Proc Natl Acad Sci U S A 108:20018–20023. 10.1073/pnas.1110416108 22128334 PMC3250195

[B14] Di Bonito M, Boulland JL, Krezel W, Setti E, Studer M, Glover JC (2015) Loss of projections, functional compensation, and residual deficits in the mammalian vestibulospinal system of *Hoxb1*-deficient mice. eNeuro 2:ENEURO.0096-15.2015. 10.1523/ENEURO.0096-15.2015 PMC4697082 26730404

[B15] Di Bonito M, Narita Y, Avallone B, Sequino L, Mancuso M, Andolfi G, Franzè AM, Puelles L, Rijli FM, Studer M (2013) Assembly of the auditory circuitry by a Hox genetic network in the mouse brainstem. PLoS Genet 9:e1003249. 10.1371/journal.pgen.1003249 23408898 PMC3567144

[B16] Díaz C, Glover JC (2002) Comparative aspects of the hodological organization of the vestibular nuclear complex and related neuron populations. Brain Res Bull 57:307–312. 10.1016/S0361-9230(01)00673-6 11922978

[B17] Díaz C, Glover JC, Puelles L, Bjaalie JG (2003) The relationship between hodological and cytoarchitectonic organization in the vestibular complex of the 11-day chicken embryo. J Comp Neurol 457:87–105. 10.1002/cne.10528 12541327

[B18] Díaz C, Puelles L, Marín F, Glover JC (1998) The relationship between rhombomeres and vestibular neuron populations as assessed in quail-chicken chimeras. Dev Biol 202:14–28. 10.1006/dbio.1998.8986 9758700

[B19] Dobin A, Davis CA, Schlesinger F, Drenkow J, Zaleski C, Jha S, Batut P, Chaisson M, Gingeras TR (2013) STAR: ultrafast universal RNA-seq aligner. Bioinformatics 29:15–21. 10.1093/bioinformatics/bts635 23104886 PMC3530905

[B20] Donevan AH, Fleming FL, Rose PK (1992) Morphology of single vestibulospinal collaterals in the upper cervical spinal cord of the cat: I. Collaterals originating from axons in the ventromedial funiculus contralateral to their cells of origin. J Comp Neurol 322:325–342. 10.1002/cne.903220304 1517483

[B21] Espana A, Clotman F (2012) Onecut transcription factors are required for the second phase of development of the A13 dopaminergic nucleus in the mouse. J Comp Neurol 520:1424–1441. 10.1002/cne.22803 22102297

[B22] Esposito MS, Capelli P, Arber S (2014) Brainstem nucleus MdV mediates skilled forelimb motor tasks. Nature 508:351–356. 10.1038/nature13023 24487621

[B23] Friese A, Kaltschmidt JA, Ladle DR, Sigrist M, Jessell TM, Arber S (2009) Gamma and alpha motor neurons distinguished by expression of transcription factor Err3. Proc Natl Acad Sci U S A 106:13588–13593. 10.1073/pnas.0906809106 19651609 PMC2716387

[B24] Giudicelli F, Gilardi-Hebenstreit P, Mechta-Grigoriou F, Poquet C, Charnay P (2003) Novel activities of Mafb underlie its dual role in hindbrain segmentation and regional specification. Dev Biol 253:150–162. 10.1006/dbio.2002.0864 12490204

[B25] Glover J (1995) Retrograde and anterograde axonal tracing with fluorescent dextran-amines in the embryonic nervous system. Neurosci Protoc 30:1–13.

[B26] Glover JC (2000a) Development of specific connectivity between premotor neurons and motoneurons in the brain stem and spinal cord. Physiolo Rev 80:615–647. 10.1152/physrev.2000.80.2.615 10747203

[B27] Glover JC (2000b) Neuroepithelial “compartments” and the specification of vestibular projections. Progr Brain Res 124:3–21. 10.1016/S0079-6123(00)24004-1 10943113

[B28] Glover JC, Petursdottir G (1988) Pathway specificity of reticulospinal and vestibulospinal projections in the 11-day chicken embryo. J Comp Neurol 270:25–38, 60–61. 10.1002/cne.902700104 3372737

[B29] Glover JC, Petursdottir G (1991) Regional specificity of developing reticulospinal, vestibulospinal, and vestibulo-ocular projections in the chicken embryo. J Neurobiol 22:353–376. 10.1002/neu.480220405 1890420

[B30] Grant GR, Farkas MH, Pizarro AD, Lahens NF, Schug J, Brunk BP, Stoeckert CJ, Hogenesch JB, Pierce EA (2011) Comparative analysis of RNA-Seq alignment algorithms and the RNA-Seq unified mapper (RUM). Bioinformatics 27:2518–2528. 10.1093/bioinformatics/btr427 21775302 PMC3167048

[B31] Grillner S, Hongo T, Lund S (1970) The vestibulospinal tract: effects on alpha-motoneurones in the lumbosacral spinal cord in the cat. Exp Brain Res 10:94–120. 10.1007/BF00340521 5411977

[B32] Gross MK, Dottori M, Goulding M (2002) Lbx1 specifies somatosensory association interneurons in the dorsal spinal cord. Neuron 34:535–549. 10.1016/S0896-6273(02)00690-6 12062038

[B33] Hamburger V, Hamilton HL (1992) A series of normal stages in the development of the chick embryo. 1951. Dev Dyn 195:231–272. 10.1002/aja.1001950404 1304821

[B34] Hempel CM, Sugino K, Nelson SB (2007) A manual method for the purification of fluorescently labeled neurons from the mammalian brain. Nat Protoc 2:2924–2929. 10.1038/nprot.2007.416 18007629

[B35] Hernandez-Miranda LR, Müller T, Birchmeier C (2017) The dorsal spinal cord and hindbrain: From developmental mechanisms to functional circuits. Dev Biol 432:34–42. 10.1016/j.ydbio.2016.10.008 27742210

[B36] Hirsch M-R, Glover JC, Dufour HD, Brunet JF, Goridis C (2007) Forced expression of Phox2 homeodomain transcription factors induces a branchio-visceromotor axonal phenotype. Dev Biol 303:687–702. 10.1016/j.ydbio.2006.12.006 17208219

[B37] Hobert O (2008) Gene regulation by transcription factors and microRNAs. Science 319:1785–1786. 10.1126/science.1151651 18369135

[B38] Huppunen J, Wohlfahrt G, Aarnisalo P (2004) Requirements for transcriptional regulation by the orphan nuclear receptor ERRgamma. Mol Cell Endocrinol 219:151–160. 10.1016/j.mce.2004.01.002 15149736

[B39] Inamata Y, Shirasaki R (2014) Dbx1 triggers crucial molecular programs required for midline crossing by midbrain commissural axons. Development 141:1260–1271. 10.1242/dev.102327 24553291

[B40] Jessell TM (2000) Neuronal specification in the spinal cord: inductive signals and transcriptional codes. Nat Rev Genet 1:20–29. 10.1038/35049541 11262869

[B41] Kang BJ, Chang DA, Mackay DD, West GH, Moreira TS, Takakura AC, Gwilt JM, Guyenet PG, Stornetta RL (2007) Central nervous system distribution of the transcription factor Phox2b in the adult rat. J Comp Neur 503:627–641. 10.1002/cne.21409 17559094

[B42] Kania A, Johnson RL, Jessell TM (2000) Coordinate roles for LIM homeobox genes in directing the dorsoventral trajectory of motor axons in the vertebrate limb. Cell 102:161–173. 10.1016/S0092-8674(00)00022-2 10943837

[B43] Kasumacic N, Glover JC, Perreault MC (2010) Segmental patterns of vestibular-mediated synaptic inputs to axial and limb motoneurons in the neonatal mouse assessed by optical recording. J Physiol 588:4905–4925. 10.1113/jphysiol.2010.195644 20962007 PMC3036187

[B44] Kasumacic N, Lambert FM, Coulon P, Bras H, Vinay L, Perreault MC, Glover JC (2015) Segmental organization of vestibulospinal inputs to spinal interneurons mediating crossed activation of thoracolumbar motoneurons in the neonatal mouse. J Neurosci 35:8158–8169. 10.1523/JNEUROSCI.5188-14.2015 26019332 PMC4444539

[B45] Kessaris N, Magno L, Rubin AN, Oliveira MG (2014) Genetic programs controlling cortical interneuron fate. Curr Opin Neurobiol 26:79–87. 10.1016/j.conb.2013.12.012 24440413 PMC4082532

[B46] Keynes R, Krumlauf R (1994) Hox genes and regionalization of the nervous system. Annu Rev Neurosci 17:109–132. 10.1146/annurev.ne.17.030194.000545 7911650

[B47] Kohl A, Marquardt T, Klar A, Sela-Donenfeld D (2015) Control of axon guidance and neurotransmitter phenotype of dB1 hindbrain interneurons by Lim-HD code. J Neurosci 35:2596–2611. 10.1523/JNEUROSCI.2699-14.2015 25673852 PMC6605615

[B48] Lambert FM, Bras H, Cardoit L, Vinay L, Coulon P, Glover JC (2016) Early postnatal maturation in vestibulospinal pathways involved in neck and forelimb motor control. Dev Neurobiol 76:1061–1077. 10.1002/dneu.22375 26724676

[B49] Lu DC, Niu T, Alaynick WA (2015) Molecular and cellular development of spinal cord locomotor circuitry. Front Mol Neurosci 8:25. 10.3389/fnmol.2015.00025 26136656 PMC4468382

[B50] Moens CB, Prince VE (2002) Constructing the hindbrain: insights from the zebrafish. Dev Dyn 224:1–17. 10.1002/dvdy.10086 11984869

[B51] Moran-Rivard L, Kagawa T, Saueressig H, Gross MK, Burrill J, Goulding M (2001) Evx1 is a postmitotic determinant of V0 interneuron identity in the spinal cord. Neuron 29:385–399. 10.1016/S0896-6273(01)00213-6 11239430

[B52] Moreno N, Bachy I, Rétaux S, González A (2005) LIM-homeodomain genes as territory markers in the brainstem of adult and developing *Xenopus laevis* . J Comp Neurol 485:240–254. 10.1002/cne.20498 15791640

[B53] Müller T, Brohmann H, Pierani A, Heppenstall PA, Lewin GR, Jessell TM, Birchmeier C (2002) The homeodomain factor lbx1 distinguishes two major programs of neuronal differentiation in the dorsal spinal cord. Neuron 34:551–562. 10.1016/s0896-6273(02)00689-x 12062039

[B54] Murray AJ, Croce K, Belton T, Akay T, Jessell TM (2018) Balance control mediated by vestibular circuits directing limb extension or antagonist muscle co-activation. Cell Rep 22:1325–1338. 10.1016/j.celrep.2018.01.009 29386118

[B55] Okaty BW, Sugino K, Nelson SB (2011) A quantitative comparison of cell-type-specific microarray gene expression profiling methods in the mouse brain. PloS One 6:e16493. 10.1371/journal.pone.0016493 21304595 PMC3029380

[B56] Ooi L, Wood IC (2008) Regulation of gene expression in the nervous system. Biochem J 414:327–341. 10.1042/BJ20080963 18717648

[B57] Pasqualetti M, Díaz C, Renaud JS, Rijli FM, Glover JC (2007) Fate-mapping the mammalian hindbrain: segmental origins of vestibular projection neurons assessed using rhombomere-specific Hoxa2 enhancer elements in the mouse embryo. J Neurosci 27:9670–9681. 10.1523/JNEUROSCI.2189-07.2007 17804628 PMC6672974

[B58] Patthey C, Clifford H, Haerty W, Ponting CP, Shimeld SM, Begbie J (2016) Identification of molecular signatures specific for distinct cranial sensory ganglia in the developing chick. Neural Dev 11:3. 10.1186/s13064-016-0057-y 26819088 PMC4730756

[B59] Pattyn A, Hirsch M, Goridis C, Brunet JF (2000) Control of hindbrain motor neuron differentiation by the homeobox gene Phox2b. Development 127:1349–1358. 10704382 10.1242/dev.127.7.1349

[B60] Pattyn A, Morin X, Cremer H, Goridis C, Brunet JF (1997) Expression and interactions of the two closely related homeobox genes Phox2a and Phox2b during neurogenesis. Development 124:4065–4075. 9374403 10.1242/dev.124.20.4065

[B61] Perreault MC, Glover JC (2013) Glutamatergic reticulospinal neurons in the mouse: developmental origins, axon projections, and functional connectivity. Ann N Y Acad Sci 1279:80–89. 10.1111/nyas.12054 23531005

[B62] Peterson BW, Coulter JD (1977) A new long spinal projection from the vestibular nuclei in the cat. Brain Res 122:351–356. 10.1016/0006-8993(77)90301-8 837234

[B63] Peterson BW, Maunz RA, Fukushima K (1978) Properties of a new vestibulospinal projection, the caudal vestibulospinal tract. Exp Brain Res 32:287–292. 10.1007/BF00239733 210030

[B64] Pierreux CE, Vanhorenbeeck V, Jacquemin P, Lemaigre FP, Rousseau GG (2004) The transcription factor hepatocyte nuclear factor-6/Onecut-1 controls the expression of its paralog Onecut-3 in developing mouse endoderm. J Biol Chem 279:51298–51304. 10.1074/jbc.M409038200 15381696

[B65] Pillai A, Mansouri A, Behringer R, Westphal H, Goulding M (2007) Lhx1 and Lhx5 maintain the inhibitory-neurotransmitter status of interneurons in the dorsal spinal cord. Development 134:357–366. 10.1242/dev.02717 17166926

[B66] Pla P, Hirsch MR, Le Crom S, Reiprich S, Harley VR, Goridis C (2008) Identification of Phox2b-regulated genes by expression profiling of cranial motoneuron precursors. Neural Dev 3:14. 10.1186/1749-8104-3-14 18565209 PMC2441621

[B67] Robinson MD, McCarthy DJ, Smyth GK (2010) edgeR: a Bioconductor package for differential expression analysis of digital gene expression data. Bioinformatics 26:139–140. 10.1093/bioinformatics/btp616 19910308 PMC2796818

[B68] Schneider CA, Rasband WS, Eliceiri KW (2012) NIH Image to ImageJ: 25 years of image analysis. Nat Methods 9:671–675. 10.1038/nmeth.2089 22930834 PMC5554542

[B69] Schubert FR, Dietrich S, Mootoosamy RC, Chapman SC, Lumsden A (2001) Lbx1 marks a subset of interneurons in chick hindbrain and spinal cord. Mech Dev 101:181–185. 10.1016/S0925-4773(00)00537-2 11231071

[B70] Shamboul KM (1980) Lumbosacral predominance of vestibulospinal fibre projection in the rat. J Comp Neur 192:519–530. 10.1002/cne.901920310 7419742

[B71] Shinoda Y, Sugiuchi Y, Izawa Y, Hata Y (2006) Long descending motor tract axons and their control of neck and axial muscles. Progr Brain Res 151:527–563. 10.1016/S0079-6123(05)51017-3 16221600

[B72] Sieber MA, Storm R, Martinez-de-la-Torre M, Müller T, Wende H, Reuter K, Vasyutina E, Birchmeier C (2007) Lbx1 acts as a selector gene in the fate determination of somatosensory and viscerosensory relay neurons in the hindbrain. J Neurosci 27:4902–4909. 10.1523/JNEUROSCI.0717-07.2007 17475798 PMC6672097

[B73] Straka H, Baker R, Gilland E (2001) Rhombomeric organization of vestibular pathways in larval frogs. J Comp Neurol 437:42–55. 10.1002/cne.1268 11477595

[B74] Studer M, Pöpperl H, Marshall H, Kuroiwa A, Krumlauf R (1994) Role of a conserved retinoic acid response element in rhombomere restriction of Hoxb-1. Science 265:1728–1732. 10.1126/science.7916164 7916164

[B75] Süsens U, Hermans-Borgmeyer I, Borgmeyer U (2000) Alternative splicing and expression of the mouse estrogen receptor-related receptor gamma. Biochem Biophys Res Commun 267:532–535. 10.1006/bbrc.1999.1976 10631096

[B76] Suwa H, Gilland E, Baker R (1996) Segmental organization of vestibular and reticular projections to spinal and oculomotor nuclei in the zebrafish and goldfish. Biol Bull 191:257–259. 10.1086/BBLv191n2p257 29220250

[B77] Tsuchida T, Ensini M, Morton SB, Baldassare M, Edlund T, Jessell TM, Pfaff SL (1994) Topographic organization of embryonic motor neurons defined by expression of LIM homeobox genes. Cell 79:957–970. 10.1016/0092-8674(94)90027-2 7528105

[B78] Uchino Y, Kushiro K (2011) Differences between otolith- and semicircular canal-activated neural circuitry in the vestibular system. Neurosci Res 71:315–327. 10.1016/j.neures.2011.09.001 21968226

[B79] Wende H, Lechner SG, Birchmeier C (2012) The transcription factor c-Maf in sensory neuron development. Transcription 3:285–289. 10.4161/trns.21809 22889842 PMC3630182

[B80] Wilson VJ, Yoshida M (1969) Comparison of effects of stimulation of Deiters’ nucleus and medial longitudinal fasciculus on neck, forelimb, and hindlimb motoneurons. J Neurophysiol 32:743–758. 10.1152/jn.1969.32.5.743 4309026

[B81] Yoshihara E, Wei Z, Lin CS, Fang S, Ahmadian M, Kida Y, Tseng T, Dai Y, Yu RT, Liddle C, Atkins AR, Downes M, Evans RM (2016) ERRγ is required for the metabolic maturation of therapeutically functional glucose-responsive β cells. Cell Metab 23:622–634. 10.1016/j.cmet.2016.03.005 27076077 PMC4832237

[B82] Zhang C, Guo ZM (2015) Multiple functions of Maf in the regulation of cellular development and differentiation. Diabetes Metab Res Rev 31:773–778. 10.1002/dmrr.2676 26122665 PMC5042042

[B83] Zhao Y, Kwan KM, Mailloux CM, Lee WK, Grinberg A, Wurst W, Behringer RR, Westphal H (2007) LIM-homeodomain proteins Lhx1 and Lhx5, and their cofactor Ldb1, control Purkinje cell differentiation in the developing cerebellum. Proc Natl Acad Sci U S A 104:13182–13186. 10.1073/pnas.0705464104 17664423 PMC1941824

