# Supplementary figures and images for: Molecular Profiling Defines Evolutionarily Conserved Transcription Factor Signatures of Major Vestibulospinal Neuron Groups
Source: eNeuro. 2019 Feb 27;6(1):ENEURO.0475-18.2019. doi: 10.1523/ENEURO.0475-18.2019 (PMC6426439; doi:10.1523/ENEURO.0475-18.2019)

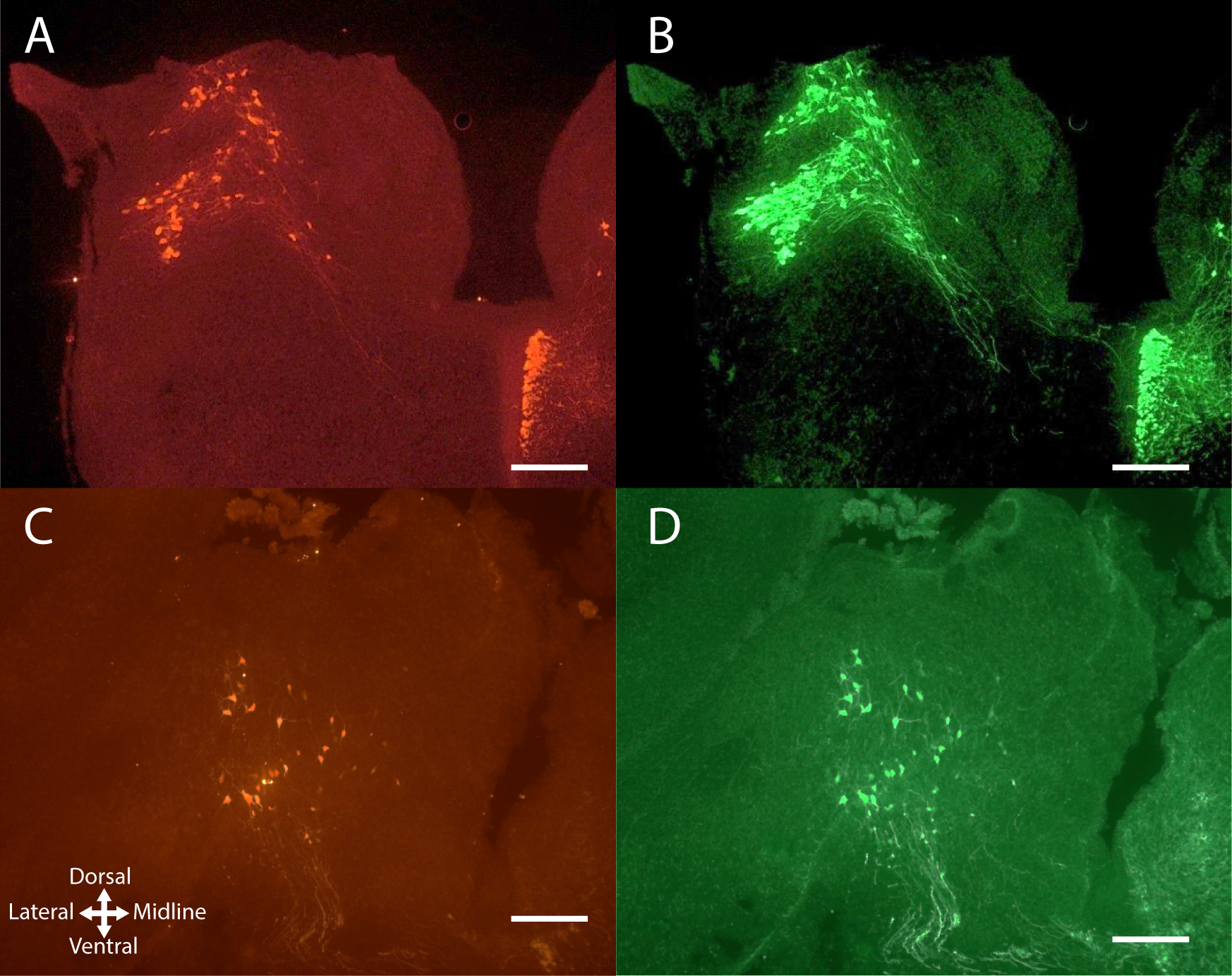

Supplement: Figure 1-1 — Retrograde labeling from mid-medulla does not label outside of the cMVST group. cMVST group in the d11 chicken embryo retrogradely labeled from C1 (A), and mid-medulla (B). cMVST group in the P1 mouse retrogradely labeled from C1 (C), and mid-medulla (D). Scale bar, 200 μm. Download Figure 1-1, TIF file. [file sup_enu-eN-NWR-0475-18-s01.tif]

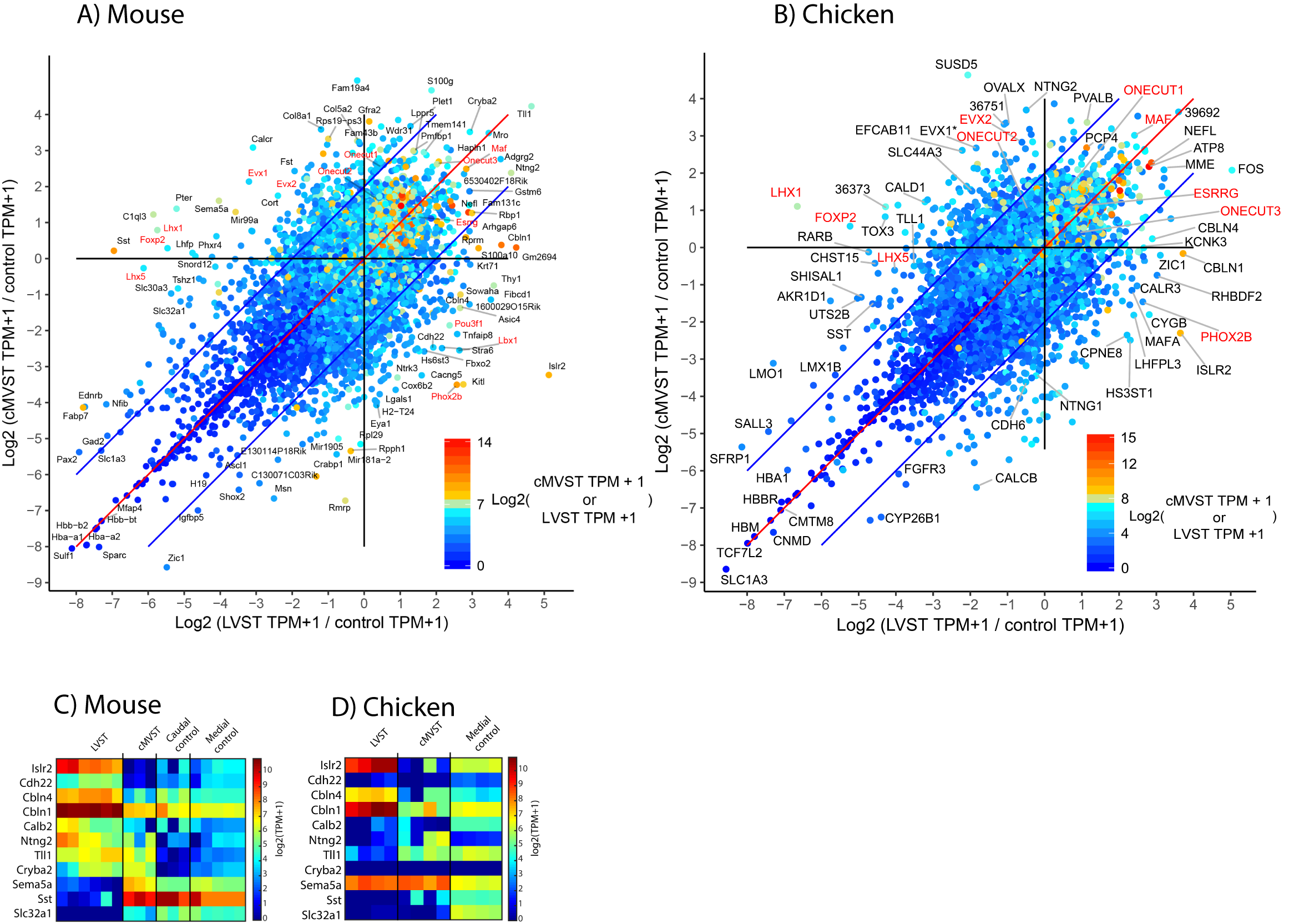

Supplement: Figure 2-1 — RNA levels and fold-changes for all transcripts in LVST versus cMVST, normalized to control tissue. All detected transcripts in mouse (A) and chicken (B) from RNAseq data (13,844 mouse and 11,982 chicken genes). A, B, Legend as in Figure 3. C, Transcript levels of select highly differentially expressed non-TF genes in individual mouse RNAseq samples. D, Transcript levels of same genes as in C for individual chicken RNAseq samples. Download Figure 2-1, TIF file. [file sup_enu-eN-NWR-0475-18-s02.tif]

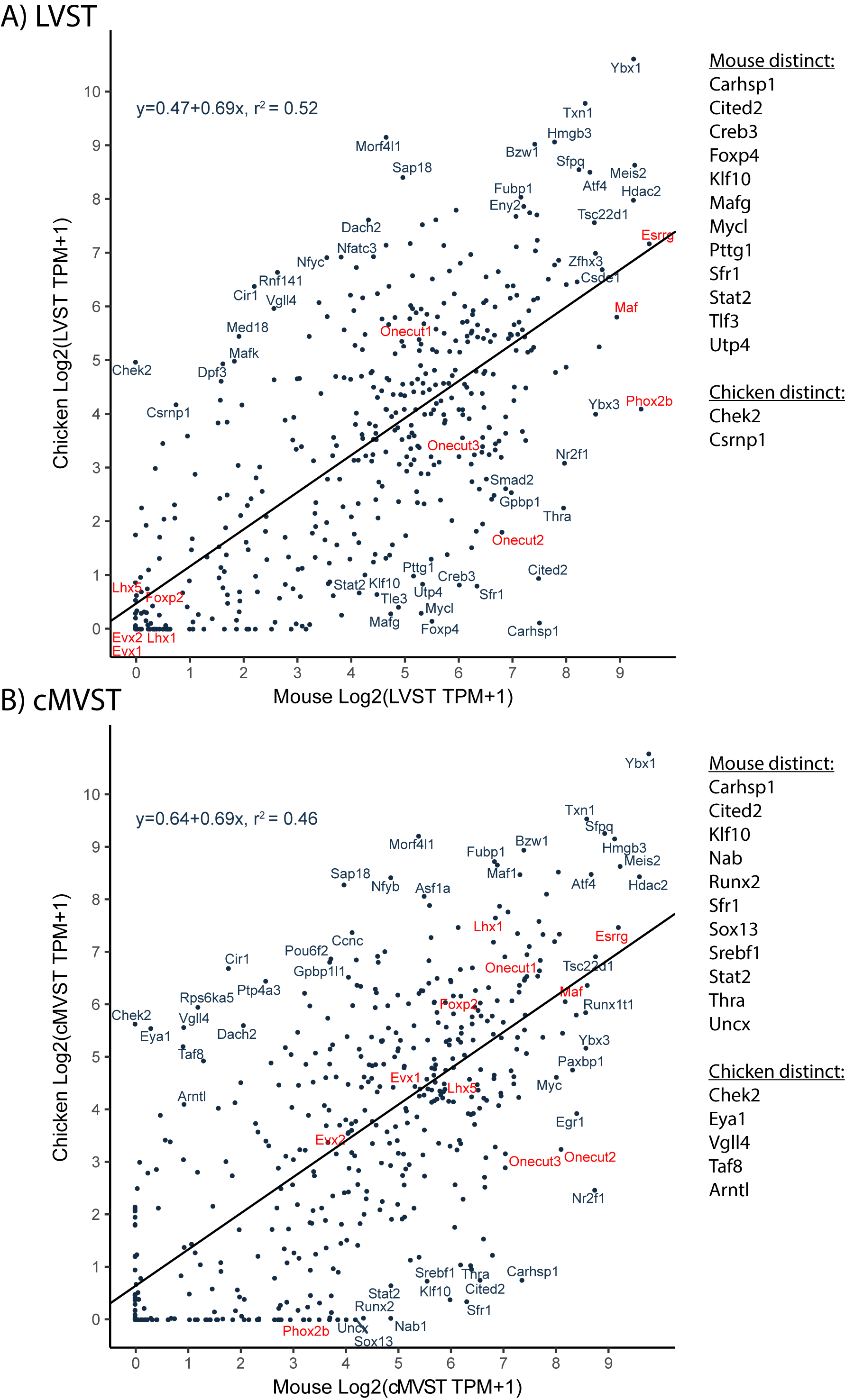

Supplement: Figure 3-1 — RNA level comparison of identified mouse/chicken TF orthologs. Plots show identified TFs that shared orthologues in mouse and chicken, plotted for Log2(TPM + 1) values, with mouse values on the x-axis and chicken values on the y-axis, for the LVST groups (A) and cMVST groups (B). Mouse-distinct TFs are defined as having Log2(TPM + 1) value >4 in mouse, and <1 in chicken, and vice versa. TF gene names (only mouse names used) shown in red indicate TFs whose expression was validated by immunohistofluorescence. Diagonal line shows the linear regression line with slope, intercept and r2 values shown at the top left in each plot. Download Figure 3-1, TIF file. [file sup_enu-eN-NWR-0475-18-s05.tif]

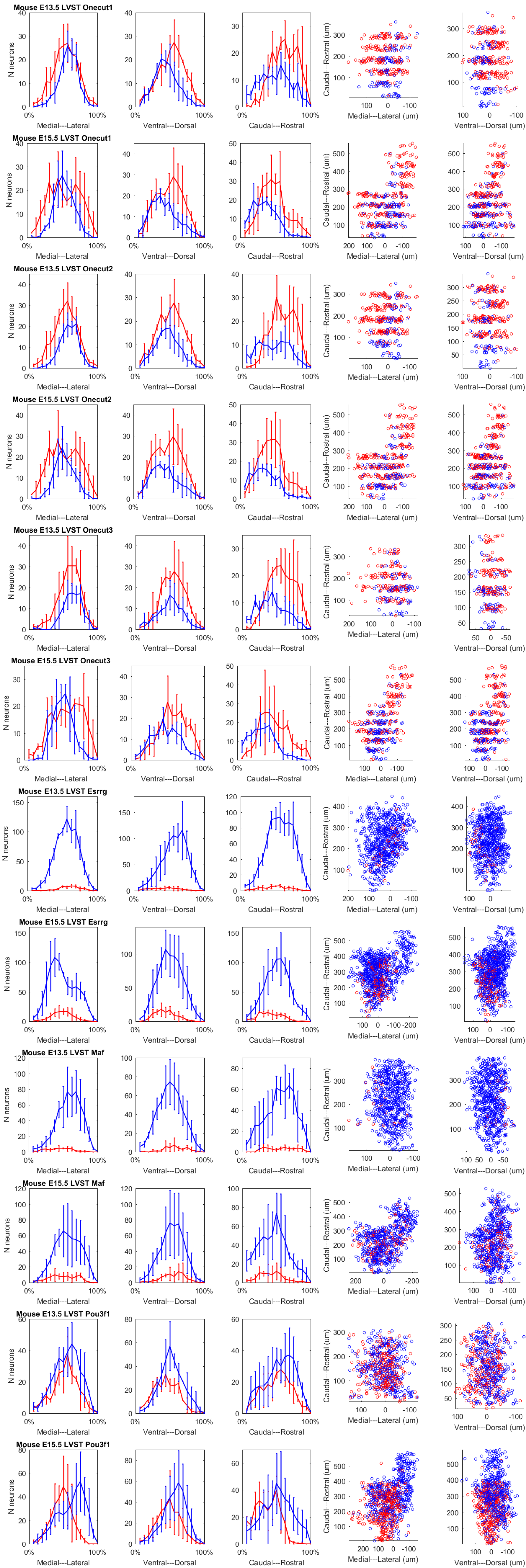

Supplement: Figure 4-1 — Histograms and scatterplots showing the spatial distribution of mouse LVST neurons expressing the indicated TFs. Neurons are indicated as either immunopositive (blue) or immunonegative (red). Histograms show averages and SD along the indicated axes. Scatterplots show projections in the indicated planes from single, representative preparations for each TF and stage. Download Figure 4-1, TIF file. [file sup_enu-eN-NWR-0475-18-s06.tif]

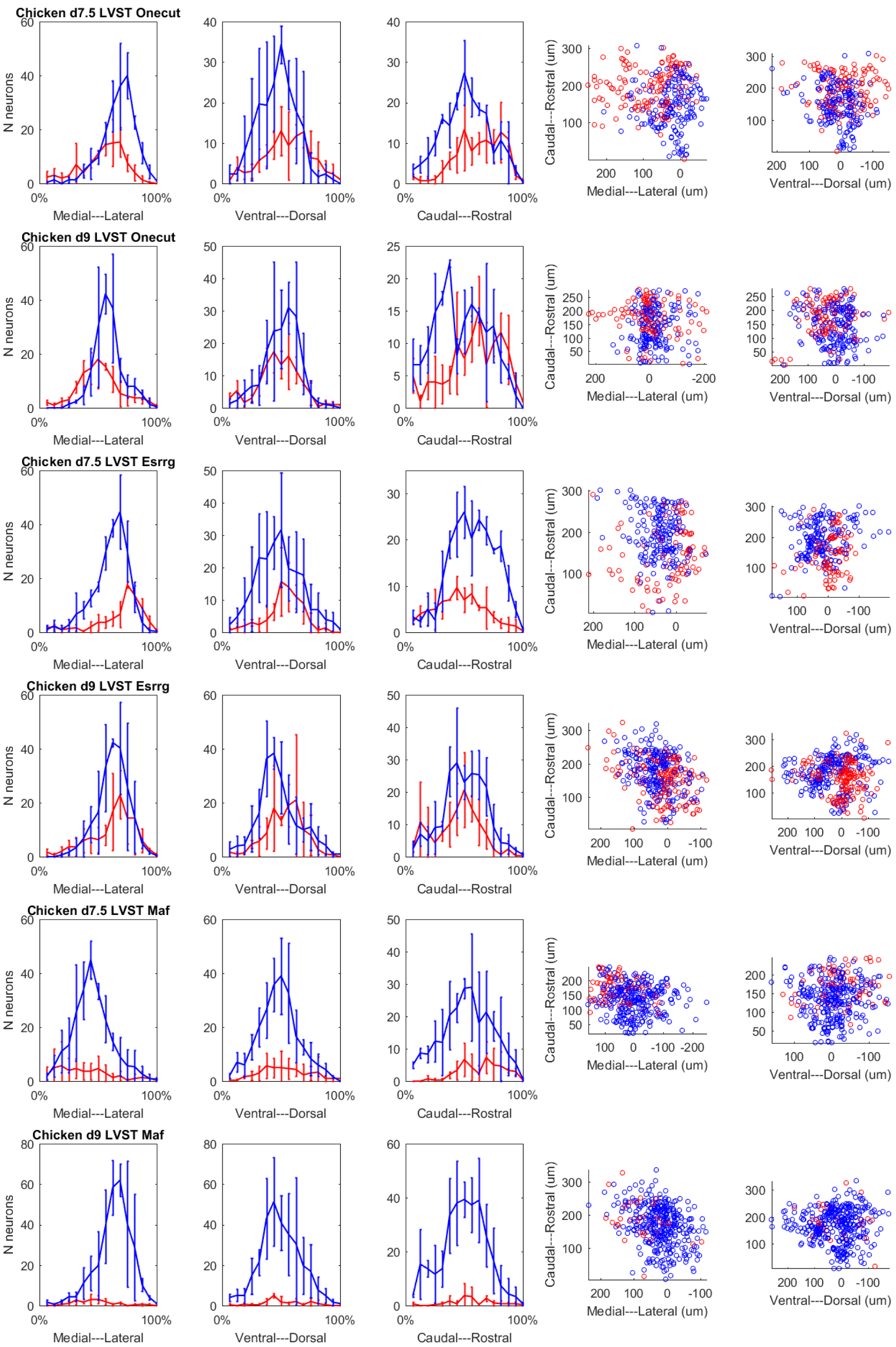

Supplement: Figure 4-2 — Histograms and scatterplots showing the spatial distribution of chicken LVST neurons expressing the indicated TFs. Legend as Figure 4-1. Download Figure 4-2, TIF file. [file sup_enu-eN-NWR-0475-18-s07.tif]

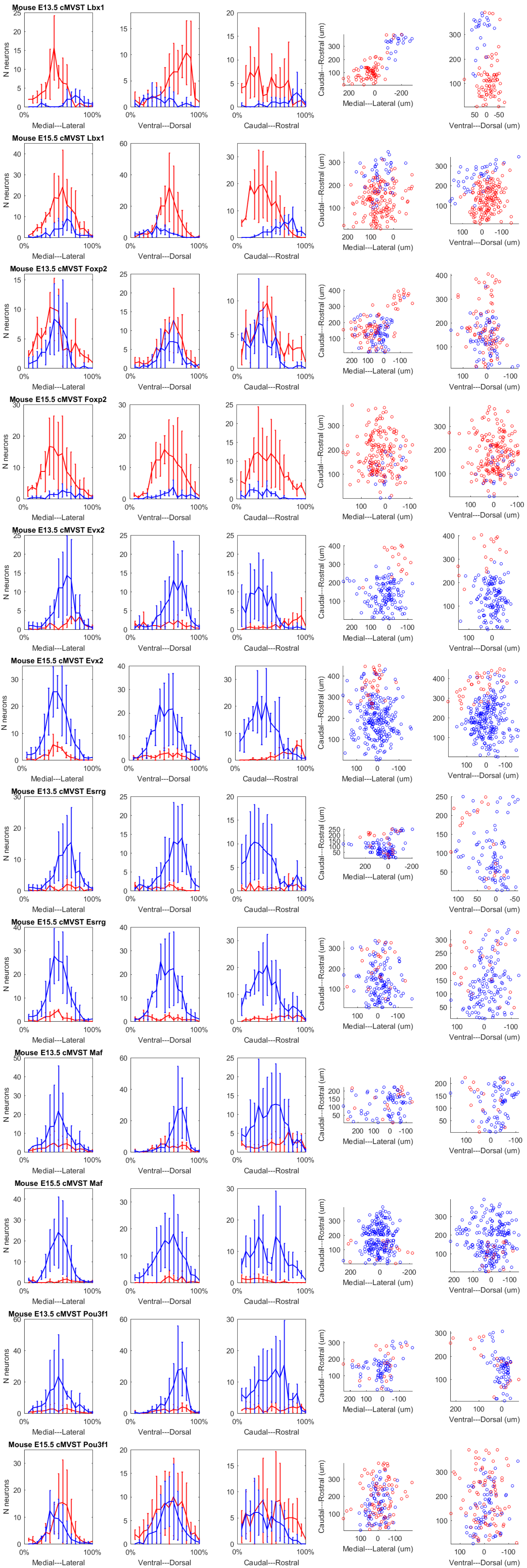

Supplement: Figure 4-3 — Histograms and scatterplots showing the spatial distribution of mouse cMVST neurons expressing the indicated TFs. Legend as Figure 4-1. Download Figure 4-3, TIF file. [file sup_enu-eN-NWR-0475-18-s08.tif]

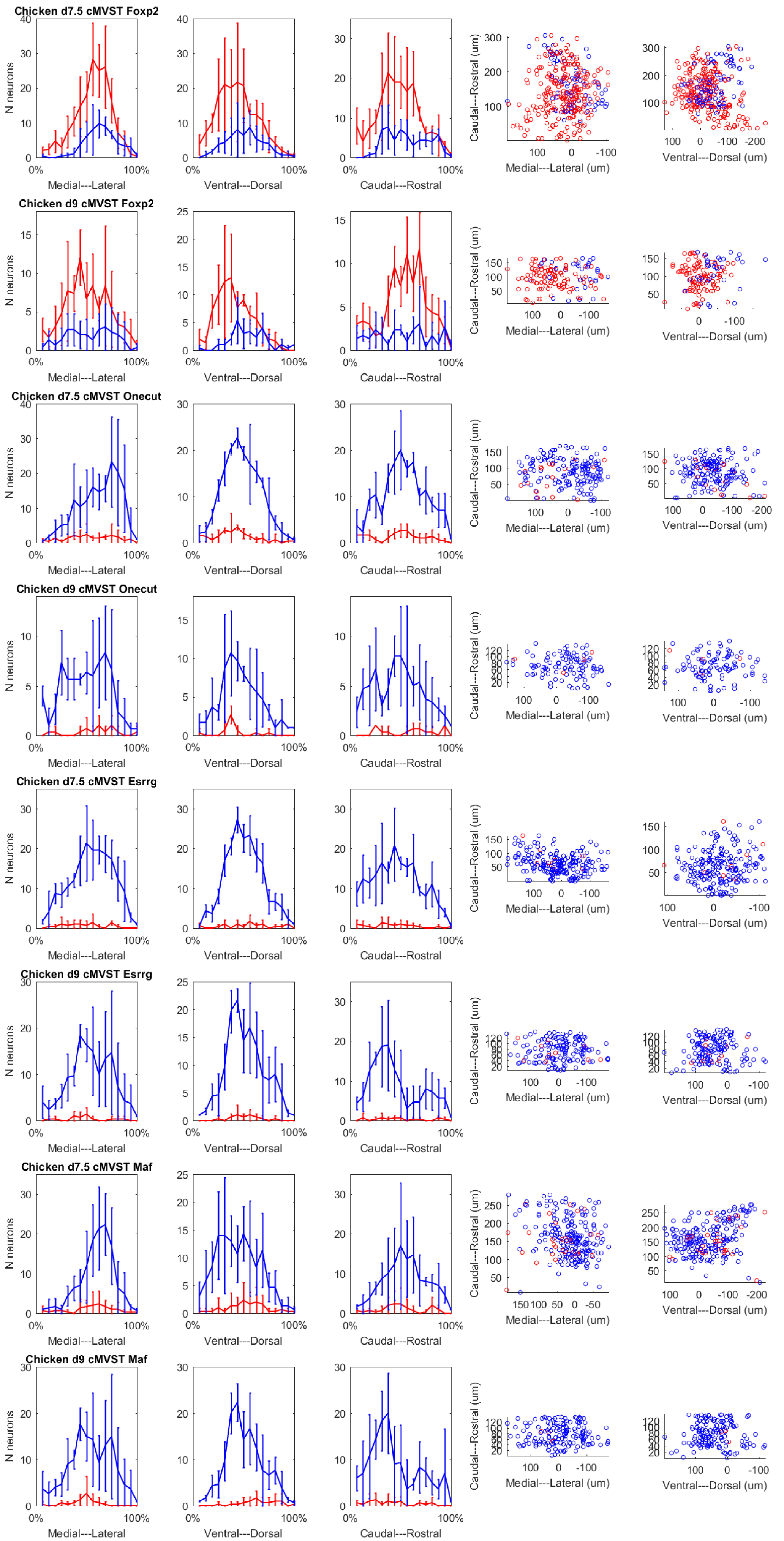

Supplement: Figure 4-4 — Histograms and scatterplots showing the spatial distribution of chicken cMVST neurons expressing the indicated TFs. Legend as Figure 4-1. Download Figure 4-4, TIF file. [file sup_enu-eN-NWR-0475-18-s10.tif]

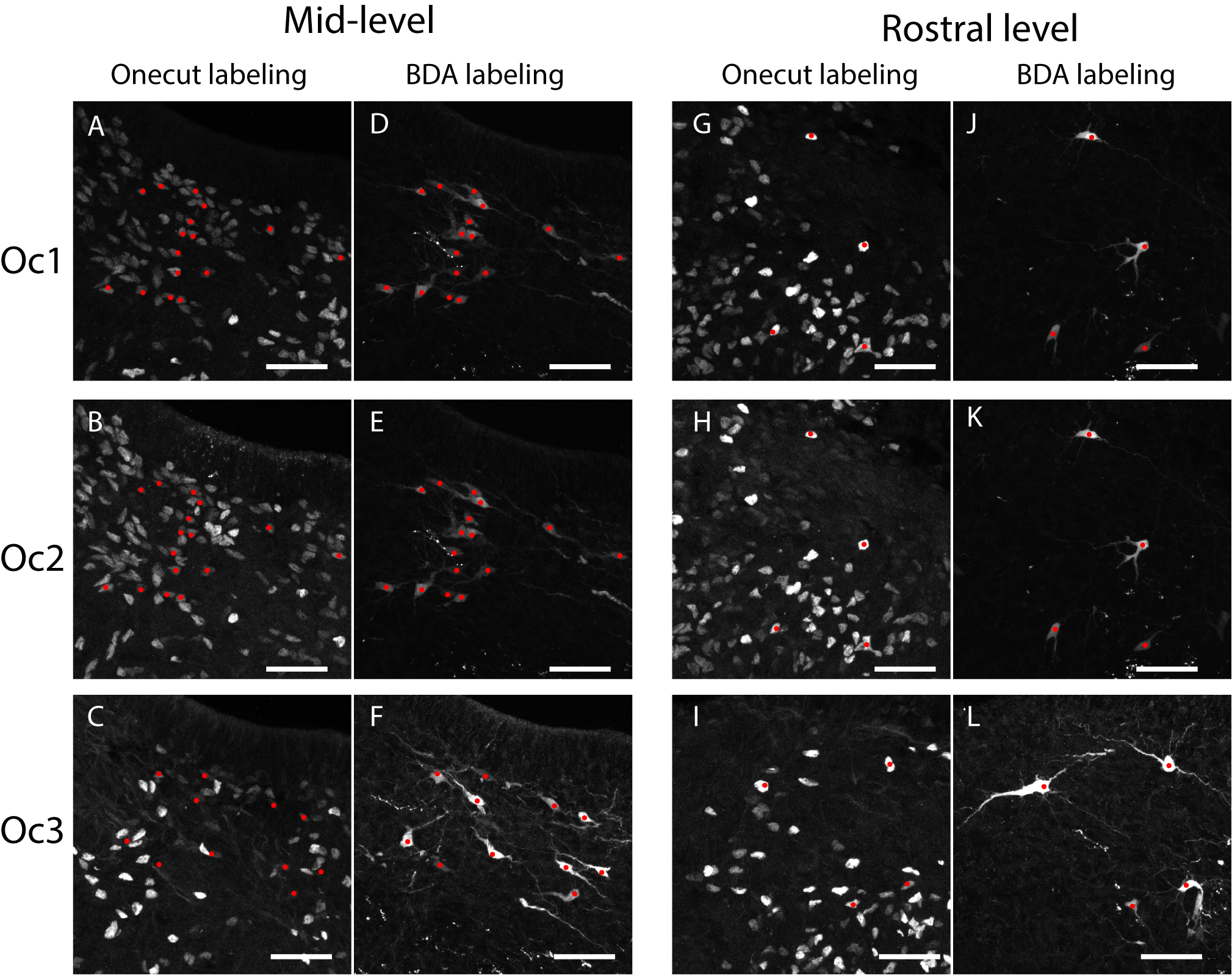

Supplement: Figure 4-6 — Differential Onecut TF staining intensity in the cMVST neuron group at different rostrocaudal levels. A–L, Confocal images of transverse sections through the E13.5 mouse cMVST, retrogradely labeled with BDA from the mid-medulla. Immunostained with antibodies specific for the indicated Onecut TFs. Imaging settings and contrast levels preserved between different rostrocaudal levels. Red dots in A–C,G–I indicate the locations of corresponding cMVST neurons in D–F,J–M. A–F, The mid rostrocaudal level of the cMVST group; G–L, the rostral level of the cMVST group. Immunostaining for Onecut TFs is stronger in rostral cMVST neurons. Dorsal, up; lateral, left; medial, right. Scale bar, 50 µm. Oc, Onecut. Download Figure 4-6, TIF file. [file sup_enu-eN-NWR-0475-18-s11.tif]
