# Supplementary material for: Molecular Profiling Defines Evolutionarily Conserved Transcription Factor Signatures of Major Vestibulospinal Neuron Groups
Source: eNeuro. 2019 Feb 27;6(1):ENEURO.0475-18.2019. doi: 10.1523/ENEURO.0475-18.2019 (PMC6426439; doi:10.1523/ENEURO.0475-18.2019)
Supplement: Figure 4-5 — Percentage of mouse LVST neurons immunopositive for a single Onecut TF. Percentage ± SD of mouse LVST neurons at indicated stages that were singly immunopositive for one (of two) Onecut factors. Oc, Onecut. Download Figure 4-5, DOCX file. [file sup_enu-eN-NWR-0475-18-s09.docx]

|  | **E13.5** | **E15.5** |
| --- | --- | --- |
| **Oc1 exclusive (vs Oc2)** | 7.2 ± 1.6 % | 5.8 ± 0.1 % |
| **Oc2 exclusive (vs O1)** | 2.3 ± 0.7 % | 2.7 ± 2.0 % |
| **Oc2 exclusive (vs Oc3)** | 3.5 ± 1.9 % | 3.3 ± 1.9 % |
| **Oc3 exclusive (vs Oc2)** | 2.1 ± 0.7 % | 4.6 ± 3.3 % |
